# Structure–Function Nexus in Calcium-Induced Polysaccharide Hydrogels: From Molecular Assembly to Texture-Tailored Geriatric Diets

**DOI:** 10.3390/foods15122210

**Published:** 2026-06-19

**Authors:** Huiqin Long, Yiqing Zhu, Gongjian Fan

**Affiliations:** 1 State Key Laboratory for Development and Utilization of Forest Food Resources, Nanjing Forestry University, Nanjing 210037, China; longhuiqin2026@163.com (H.L.); yiqing.zhu@njfu.edu.cn (Y.Z.); 2 Co-Innovation Center for the Sustainable Forestry in Southern China, Nanjing Forestry University, Nanjing 210037, China

**Keywords:** calcium ion, polysaccharide gel, crosslinking mechanism, geriatric food

## Abstract

Calcium-induced polysaccharide hydrogels have attracted growing interest in food science because of their mild gelation conditions, tunable structures, and compatibility with food-grade formulation. This review focuses on edible Ca^2+^-mediated polysaccharide hydrogels and related composite networks, focusing on alginate, low-methoxyl pectin, gellan gum, and carrageenan. Rather than treating all calcium-containing polysaccharide materials as well-defined complexes, we distinguish direct coordination, ionic bridging, charge screening, helix stabilization, and composite-assisted network regulation. Current evidence indicates that Ca^2+^-mediated assembly is governed by polysaccharide fine structure, calcium-release behavior, pH, ionic strength, and processing conditions, thereby determining crosslinking density, digestibility gel strength, water distribution, rheological properties, release behavior, and texture-related functionality. For texture-modified foods for older adults, these hydrogels may provide a useful material basis for designing swallowing-friendly matrices, sustained nutrient-delivery systems, and soft composite foods. However, available evidence is still largely derived from model gels, in vitro characterization, and static digestion models, while validation in real food matrices, dynamic gastrointestinal conditions, oral processing, sensory acceptance, and older-adult populations remains limited. Future studies should establish structure–function–population evidence chains linking molecular assembly to reliable geriatric food performance.

## 1. Introduction

Polysaccharides are natural macromolecules with diverse sources, structural heterogeneity, and good biocompatibility, and they are widely used as food hydrocolloids, stabilizers, texture modifiers, and delivery matrices. Among them, anionic polysaccharides containing carboxyl or sulfate groups, such as alginate, pectin, carrageenan, and gellan gum, can interact with calcium ions (Ca^2+^) through specific or nonspecific ionic associations to form three-dimensional hydrogel networks [1]. The classical “egg-box” model has provided a fundamental framework for understanding Ca^2+^-induced gelation, especially in alginate and low-methoxyl pectin (LMP) systems [2]. However, calcium-mediated gelation in food-relevant polysaccharide systems is not limited to a single binding mode. Depending on polysaccharide fine structure, charge density, calcium source, pH, ionic strength, and processing conditions, Ca^2+^ may contribute to network formation through direct coordination, ionic bridging, charge screening, helix stabilization, or composite-assisted regulation [3,4,5].

With the increasing complexity of food matrices, Ca^2+^-induced polysaccharide hydrogels have gradually evolved from single-component model systems to multicomponent networks involving polysaccharide–polysaccharide, polysaccharide–protein, and polysaccharide–starch interactions. In these composite polysaccharide-based systems, Ca^2+^ can function not only as a crosslinking ion but also as a molecular bridge or network regulator, mediating both synergistic and competitive interactions among biopolymers [3,4,5]. Such interactions determine gelation kinetics, crosslinking density, pore architecture, hydration state, rheological behavior, and mechanical stability, which together govern food-relevant functions such as encapsulation, controlled release, texture modulation, barrier protection, and digestive behavior. Therefore, understanding the structure–function relationship of calcium-induced polysaccharide hydrogels is essential for translating molecular assembly principles into rational food design.

Population aging has further increased the demand for texture-modified and nutrition-oriented foods. Older adults frequently experience physiological changes such as impaired mastication and swallowing, reduced digestive capacity, and altered intestinal microecology [6,7]. Dysphagia is particularly relevant to food texture design because it increases the risk of aspiration, insufficient food intake, and malnutrition [8]. Consequently, foods for older adults should not only provide nutrients but also meet requirements related to swallowing safety, texture consistency, nutrient delivery, and gastrointestinal tolerance [9,10,11]. Calcium-induced polysaccharide hydrogels are attractive in this context because their structures and textural properties can be adjusted by controlling polysaccharide composition, Ca^2+^ release behavior, and processing conditions.

Although numerous studies have investigated Ca^2+^-induced gelation of individual polysaccharides or specific composite polysaccharide-based systems, several limitations remain. First, many studies describe calcium-containing polysaccharide materials as “complexes” without clearly distinguishing direct coordination, ionic crosslinking, electrostatic screening, and secondary network regulation. Second, existing reports often emphasize isolated properties such as gel strength, encapsulation efficiency, or release behavior, while the causal chain from molecular assembly to multiscale structure and final food functionality is not sufficiently integrated. Third, differences in polysaccharide source, molecular weight, degree of esterification or amidation, block distribution, calcium salt type, and evaluation method make it difficult to compare results across studies [12,13]. Finally, evidence supporting applications in foods for older adults is still largely based on model gels, in vitro characterization, and static digestion simulations, whereas validation in real food matrices, oral processing, sensory acceptance, dynamic gastrointestinal models, and older-adult populations remains limited.

Compared with previous studies that mainly focus on calcium-induced gelation mechanisms, individual polysaccharide systems, or specific delivery applications, this review aims to provide an integrated structure–function framework for edible Ca^2+^-mediated polysaccharide hydrogels and related composite networks. The novelty of this review lies in three aspects. First, it distinguishes different roles of Ca^2+^ in food polysaccharide systems, including direct coordination, ionic bridging, charge screening, and composite-assisted network regulation. Second, it links molecular assembly with multiscale structural features and functional outputs, thereby clarifying how crosslinking density, polysaccharide fine structure, hydration state, and pore architecture affect texture, release, digestion, and probiotic protection. Third, it evaluates the potential and evidence boundaries of these hydrogels in texture-modified foods for older adults, with attention to swallowing-friendly matrices, sustained nutrient delivery, and intestinal health support.

Accordingly, this review first summarizes the Ca^2+^-mediated assembly mechanisms in single and composite polysaccharide-based systems. It then discusses multiscale characterization strategies for revealing network morphology, intermolecular interactions, hydration state, and thermal stability. In particular, rheological characterization is considered as a critical component of this multiscale framework because calcium-induced polysaccharide hydrogels are highly hydrated, deformable, and time-dependent food structures. Unlike static imaging or spectroscopic analyses, rheology directly evaluates gelation kinetics, viscoelastic balance, yield behavior, flow stability, thixotropic recovery, and temperature-dependent mechanical responses. These parameters provide essential evidence for determining whether a molecularly assembled network can function as a stress-bearing food matrix and whether it can meet application-related requirements such as texture control, oral processing, swallowing suitability, 3D printability, and digestive stability. Subsequently, the review analyzes the major food-related functions of calcium-induced polysaccharide hydrogels, including encapsulation and controlled release, texture regulation, barrier protection, starch digestibility modulation, and probiotic delivery. Beyond material-level performance, this review also considers how calcium-induced polysaccharide hydrogels can be evaluated within a translational framework for geriatric food design. Model gels, static digestion assays, and isolated texture measurements can demonstrate structural feasibility, but they cannot fully establish real eating effectiveness. Therefore, attention is given to the need for real-food validation, dynamic gastrointestinal models, oral-processing assessment, sensory evaluation, repeated-intake acceptance, and older-adult-relevant outcomes. Finally, it discusses their application prospects in texture-modified foods for older adults and highlights the methodological gaps that must be addressed before laboratory findings can be translated into reliable food products.

## 2. Mechanism of Calcium Ion-Induced Polysaccharide Gel Formation

### 2.1. Crosslinking Mechanisms in Single-Polysaccharide Systems

In single-polysaccharide systems, Ca^2+^-induced gelation should not be interpreted simply as “gel formation after calcium addition”. Instead, it depends on the type, density, spatial arrangement, and accessibility of binding sites along the polysaccharide chains. For anionic polysaccharides with continuous carboxylate or sulfate domains, Ca^2+^ can act as an effective ionic crosslinking node; for polysaccharides dominated by hydroxyl groups or discontinuous charge distributions, it more often functions through charge screening, weak complexation, or structural modulation. Therefore, single-polysaccharide systems should first be distinguished as strong direct-coordination systems or weak regulation systems, rather than being uniformly assigned to the egg-box model. This distinction provides the basis for interpreting subsequent structure–function relationships.

Low-methoxyl pectin and alginate are the most representative strong direct-coordination systems, but their structural determinants differ. Natural pectin consists mainly of homogalacturonan, rhamnogalacturonan I (RG-I), and rhamnogalacturonan II (RG-II) domains [14,15], and low-methoxyl pectin forms ordered Ca^2+^-mediated junction zones primarily through free carboxyl groups in the homogalacturonan (HG) regions [16,17,18]. Thermodynamic and simulation studies further suggest that pectin–Ca^2+^ association is not a static one-step event, but may involve a transition from initial single-site binding to ordered multipoint crosslinking [2]. On this basis, the gelation capacity of LMP is controlled not only by the number of carboxyl groups but also by HG continuity, RG-I branching, and molecular weight [12,19]. Thus, LMP-Ca^2+^ gels should be viewed as systems jointly governed by binding-site availability and chain-level assembly capacity.

High-methoxyl pectin (HMP) provides an important counterexample. Because of its limited number of free carboxyl groups, HMP is less able to form continuous egg-box junction zones; Ca^2+^ mainly weakens electrostatic repulsion, promotes chain aggregation, and reinforces weak-gel behavior [20]. Comparative studies show that decreasing the degree of esterification increases Ca^2+^ responsiveness and enhances gelation capacity [13,20]. Studies on beet pulp pectin and potato pectin further indicate that even within pectin systems, Ca^2+^-induced gelation may shift among egg-box crosslinking, ionic bridging, and hydrogen-bond-dominated structures [17,18]. Therefore, “pectin–calcium gel” should not be treated as a single mechanistic label, but as a structure-dependent family governed by degree of esterification (DE), HG/RG-I ratio, and botanical origin.

Chemical modification further demonstrates the designability of pectin–Ca^2+^ systems. Zhang et al. reduced methyl ester groups in pectin and improved its Ca^2+^ responsiveness, allowing gelation without additional sucrose and increasing gelation temperature with modification degree [21]. Wei et al. prepared amidated low-ester pectins with different degree of amidation (DA) and DE values and found that moderate amidation strengthened the network through additional hydrogen bonding, whereas excessive amidation caused pre-gelation and reduced water-holding capacity [22]. These studies show that stronger Ca^2+^ responsiveness does not necessarily mean better gel performance. A more appropriate interpretation is that pectin modification requires a balance among binding-site density, network homogeneity, and water retention.

Alginate also follows the egg-box model, but its key determinant is the content and distribution of guluronic acid blocks. Ca^2+^ preferentially coordinates with α-L-guluronic acid regions to form high-affinity junction zones, whereas mannuronic acid blocks (M) and alternating guluronic acid/mannuronic acid block (GM) regions mainly affect flexibility and elasticity [1,9]. Thus, a high guluronic acid block (G-block) content generally favors strong but brittle networks, while higher M or GM proportions may generate softer mechanical responses. Compared with pectin, alginate offers clearer crosslinking rules, a wider gelation window, and higher operational tunability, but excessive crosslinking may increase brittleness and restrict release.

Alginate systems also highlight the importance of Ca^2+^-release kinetics. Internal gelation using calcium ethylenediaminetetraacetate (Ca-EDTA) or poorly soluble calcium salts under acid-triggered release can reduce structural gradients caused by rapid external diffusion [23,24]. Crosslinking density and aging time alter pore size, wall thickness, and water state, thereby affecting microbial degradation and short-chain fatty acid release [9,25]. Lin et al. compared rapid alginate gelation induced by different calcium sources using real-time rheology and found that calcium chloride (CaCl_2_) produced the fastest gelation kinetics [26]. Wang et al. showed that anion type can regulate pore structure and thermal stability by modifying hydration, especially in alginates with a high mannuronic acid/guluronic acid ratio (M/G ratios) [27]. These findings indicate that alginate gel design should report not only Ca^2+^ concentration but also calcium source, release rate, and ionic environment.

Compared with carboxylate-rich polysaccharides, sulfate-containing polysaccharides rely more strongly on helix formation and chain aggregation. In κ-carrageenan (KC), Ca^2+^ can form multidentate interactions with sulfate groups, hydroxyl groups, and cyclic ether oxygen, while hydrogen bonding jointly promotes the coil-to-helix transition and helix aggregation [28]. In ι-carrageenan (ι Car), Ca^2+^ mainly stabilizes gel coatings through sulfate-associated ionic bridging and has been used for nanoparticle encapsulation [29]. Therefore, carrageenan–calcium systems should not be equated with alginate or LMP egg-box networks. A more accurate description is that Ca^2+^ reinforces gelation by stabilizing charged helical aggregates rather than by simply constructing carboxylate junction boxes.

Starch represents a different weak-interaction system. Because starch lacks continuous anionic binding domains, Ca^2+^ mainly affects gelatinization, crystallinity, and digestibility through hydroxyl-related weak complexation, electrostatic interactions, van der Waals forces, or processing-induced structural rearrangement. Ca^2+^ released from calcium hydroxide nanoparticles can weakly interact with amylose and amylopectin in corn starch and increase system viscosity [30], while Ca^2+^ chelation with hydroxyl groups in black soybean resistant starch increases retrograded resistant starch (RS3) content [31]. Ultra-high-pressure treatment further changes the ordering and mobility of starch–calcium complexes; 200 MPa enhances structural order and resistant starch content, whereas 500 MPa may disrupt crystalline organization [32]. Therefore, starch–calcium systems are better considered as digestion-modulating and texture-assisting systems rather than typical ionically crosslinked hydrogels.

Some naturally derived plant polysaccharides further show that Ca^2+^ responsiveness is influenced by monosaccharide composition and endogenous mineral states. In Ulva polysaccharides, Ca^2+^ forms ionic crosslinks with glucuronic acid carboxyl groups, leading to network densification and increased gel strength with increasing Ca^2+^ concentration [33]. Nigella sativa polysaccharide behaves differently, as its gelation is mainly driven by hydrogen bonding and endogenous Ca^2+^ may even inhibit network formation [34]. Blueberry pomace polysaccharide forms a dense network at 15 mmol/L Ca^2+^ through electrostatic attraction, hydrogen bonding, and hydrophobic interactions, whereas excessive Ca^2+^ weakens gel formation [35]. Premna microphylla Turcz polysaccharide interacts electrostatically with Ca^2+^, increasing storage modulus, gel strength, and thermal stability [36]. These cases suggest that naturally sourced polysaccharides often have an optimal Ca^2+^ window, and their behavior cannot be directly extrapolated from alginate or pectin models.

**Figure 1:**
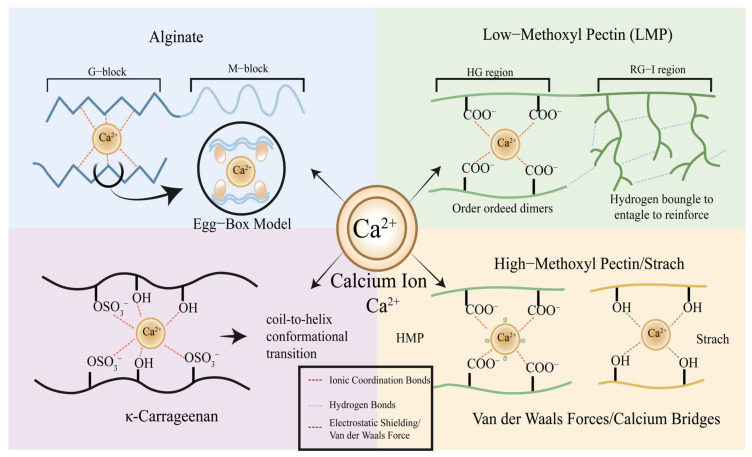
Crosslinking mechanisms of Ca^2+^ in single-polysaccharide systems.

The main Ca^2+^-mediated assembly modes in single-polysaccharide systems are summarized in Figure 1. Rather than representing all systems by a single egg-box model, the figure highlights a mechanistic continuum from strong ionic crosslinking to weak structural regulation.

Although single-polysaccharide systems provide the basic mechanistic models for Ca^2+^-induced gelation, food matrices usually contain multiple biopolymers. In such systems, Ca^2+^ may no longer act only as a primary crosslinking ion, but may also mediate component competition, network coupling, and phase organization.

### 2.2. Crosslinking Mechanisms in Composite Polysaccharide-Based Systems

Compared with single-polysaccharide systems, composite polysaccharide-based systems do not involve Ca^2+^ merely as a well-defined ionic crosslinking node. Here, the term composite polysaccharide-based systems refers to networks in which polysaccharides serve as the main Ca^2+^-responsive phase and interact with other polysaccharides, proteins, starches, lipids, or particulate fillers. In these systems, Ca^2+^ acts not only as a primary ionic crosslinking ion, but may also mediate component competition, network synergy, phase organization, and structural rearrangement. Its final effect depends on the Ca^2+^ affinity of different biopolymers, chain conformation, mixing sequence, calcium-release kinetics, pH, and ionic strength. Therefore, in composite polysaccharide-based systems, Ca^2+^ should be regarded more as a network-regulating factor than as a simple crosslinker. This view helps avoid reducing all composite gels to reinforced egg-box structures.

The first mechanism is competitive binding, which typically occurs when two components share the same or similar anionic binding sites. In sodium alginate (SA)/chitosan oligosaccharide systems, Ca^2+^ and chitosan oligosaccharide (COS) compete for carboxylate sites on alginate chains; increasing Ca^2+^ enhances mechanical strength but weakens COS surface association and related functionality [37]. A similar competitive relationship was observed in SA/COS beads loaded with krill oil, where the balance between Ca^2+^ and COS directly affected oil loading and oxidative stability [38]. Thus, competition is not necessarily a drawback, but a formulation variable for balancing mechanical strength, interfacial composition, and functional loading.

The second mechanism is the formation of an interpenetrating network composed of one ionic network and one physically entangled secondary network. In alginate/konjac glucomannan (KGM) systems, Ca^2+^ preferentially crosslinks alginate G-blocks, whereas KGM mainly forms a secondary network through chain entanglement and hydrogen bonding [39]. In lotus rhizome starch/sodium alginate systems, Ca^2+^ crosslinks alginate as the primary ionic framework, while gelatinized or dextrinized starch contributes through hydrogen bonding and physical filling [10]. This mode can improve water retention and structural integrity without greatly increasing ionic crosslinking strength, but its stability depends strongly on the compatibility between the secondary component and the primary network.

The third mechanism involves true multiple ionic crosslinking, in which Ca^2+^ participates in the network formation of two or more polymers. In κ-carrageenan/carboxymethylcellulose double-crosslinked hydrogels, Ca^2+^ interacts with sulfate groups of κ-carrageenan and carboxyl groups of carboxymethylcellulose (CMC), while hydrogen bonding further contributes to a denser network [4]. In low-acyl gellan gum/low-methoxyl citrus pectin co-gels, the gellan junction zones appear dominant, whereas pectin is further reinforced by calcium bridges and hydrogen bonds [40]. In oxidized sodium alginate/chitosan hydrogels, Schiff-base bonding and Ca^2+^–carboxylate coordination jointly form a double-crosslinked network, leading to higher hardness and adsorption capacity with increasing Ca^2+^ concentration [41]. Such systems can improve strength and compactness, but they are also more vulnerable to incompatibility, local over-crosslinking, and network heterogeneity.

It should be emphasized that Ca^2+^ reinforcement does not necessarily mean performance optimization. In cellulose nanocrystal-modified agar films, moderate Ca^2+^ crosslinking produces a dense and smooth surface, whereas excessive Ca^2+^ causes roughness and aggregation [42]. In carboxymethylcellulose/gelatin composite films, Ca^2+^ concentration, temperature, and humidity jointly affect surface pore structure, and excessive crosslinking may generate structural defects [43]. Therefore, the key objective in composite polysaccharide-based systems is not to maximize crosslinking density, but to identify an optimal window that balances strength, homogeneity, water retention, and processing stability.

In polysaccharide–protein composite polysaccharide-based systems, Ca^2+^ often functions as a cationic bridge connecting negatively charged groups on polysaccharide and protein surfaces. Fourier transform infrared spectroscopy (FTIR) and ζ-potential analyses of pea protein isolate (PPI)/high-methoxyl pectin systems indicate that Ca^2+^-mediated electrostatic interactions are a major driving force for complex formation [5]. In zein nanoparticle/ι-carrageenan systems, a layer-by-layer strategy first promotes adsorption and then uses Ca^2+^ crosslinking to solidify a dense gel shell, thereby improving encapsulant stability [29]. In pectin-based emulsion gels, Ca^2+^-crosslinked pectin emulsion gels showed a denser structure and stronger digestive resistance, supporting colon-targeted release [44]. Compared with direct simultaneous mixing, sequential assembly can reduce uncontrolled aggregation and local crosslinking, making it a more controllable strategy for delivery-system construction.

However, in protein–polysaccharide or starch–protein composite polysaccharide-based systems, Ca^2+^ does not always improve gel strength. In mung bean starch/flaxseed protein gels, calcium salts promote protein aggregation and amylose–protein crosslinking, but they also alter amylose conformation and finally generate weaker gel networks [6]. In rice starch/soy protein systems, calcium citrate and calcium carbonate improve gel performance by increasing pH and promoting β-sheet-rich bicontinuous networks, whereas calcium gluconate and calcium chloride lower pH and lead to disordered aggregation [45]. In mung bean protein/gellan gum systems, low-acyl gellan gum and proteins show both synergistic interactions and Ca^2+^ competition, resulting in nonlinear changes in gel properties with Ca^2+^ concentration [46]. In sorghum bran arabinoxylan/soy protein isolate (SPI) gels, low Ca^2+^ levels strengthen the network, but concentrations above a threshold disrupt the structure and reduce elasticity and chewability [47]. These findings indicate that Ca^2+^ can act either as a structural enhancer or as a trigger for phase separation and excessive aggregation.

In ternary or multicomponent systems, Ca^2+^ functions more like a network coupler. In fava bean 11S nanofiber/κ-carrageenan gels, Ca^2+^ forms calcium bridges with carboxyl groups on protein fibers and coordinates with sulfate groups on κ-carrageenan, while electrostatic attraction and hydrogen bonding also occur between the two biopolymers [48]. Such systems can integrate rigid networks, flexible filling, and interfacial interactions, but they are also the most susceptible to oversimplified mechanistic interpretation. For multicomponent gels, the key question is not whether Ca^2+^ participates in crosslinking, but how much each interaction contributes to mechanics, pore structure, and release behavior.

Composite polysaccharide-based systems also highlight dynamic regulation in the time dimension. Wang et al. used glucono-δ-lactone (GDL) and poorly soluble calcium salts to induce protein acid gelation first, followed by gradual Ca^2+^ release and alginate crosslinking, ultimately forming an interpenetrating network with improved performance [49]. This sequential gelation strategy transforms Ca^2+^ crosslinking from an instantaneous reaction into a programmable dynamic process. Its main value lies not simply in increasing gel strength, but in reducing structural heterogeneity through controlled reaction sequencing.

In real food or processing systems, Ca^2+^ often contributes simultaneously to spatial support, network filling, and processing adaptability. In corn-starch-based 3D-printing inks, Ca^2+^ forms ionic bridges with anionic hydrocolloids and also interacts with gelatinized starch through hydrogen bonding and van der Waals forces [7]. In pectin gel beads, starch derivatives increase internal crosslinking and generate a more compact porous structure [50]. In konjac glucomannan films, the crosslinking ability of different metal ions follows the order Fe^3+^ > Cu^2+^ > Ca^2+^, indicating that Ca^2+^ is not always the strongest structure-regulating ion [51]. In SA–pectin–whey protein wall systems, Ca^2+^ crosslinks both alginate and pectin, while whey protein fills network voids to form a dense structure [52]. These examples show that Ca^2+^ action in real food matrices is usually a composite effect determined jointly by formulation components and processing pathways.

**Figure 2:**
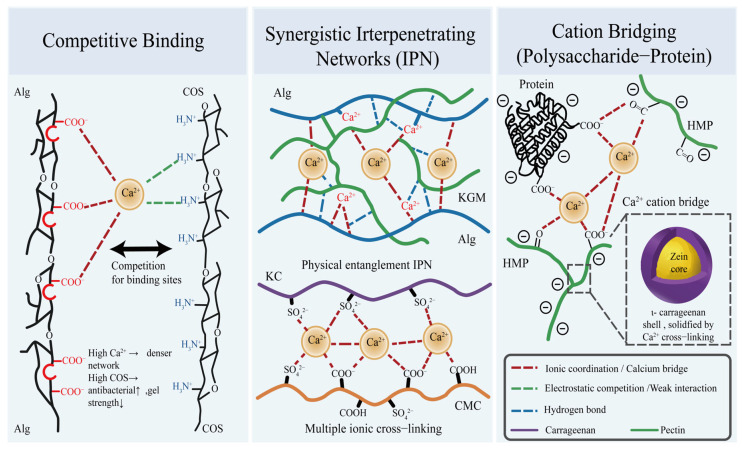
Ca^2+^-mediated network regulation in composite polysaccharide-based systems.

The roles of Ca^2+^ in composite polysaccharide-based systems are summarized in Figure 2: Ca^2+^-mediated network regulation in composite polysaccharide-based systems. Compared with single-polysaccharide gels, composite polysaccharide-based systems require a broader interpretation of Ca^2+^ as a competing ion, molecular bridge, sequential gelation trigger, or network-regulating factor.

These mechanistic models explain how Ca^2+^ can initiate or regulate hydrogel formation, but they do not directly reveal the resulting network architecture. Therefore, multiscale characterization is required to connect molecular assembly with observable structural features, including morphology, intermolecular interactions, hydration state, and thermal stability.

## 3. Structural Characterization of Calcium Ion-Induced Polysaccharide Gels

### 3.1. Imaging of Network Morphology

Network imaging mainly addresses four questions: whether Ca^2+^ induces a continuous network, whether the network becomes denser with smaller pores, thicker pore walls, or a more homogeneous pore-size distribution, whether phase separation occurs in composite polysaccharide-based systems, and whether active compounds or microorganisms are uniformly distributed. Imaging provides direct spatial evidence, but it usually cannot identify Ca^2+^ coordination sites, binding strength, or stoichiometry. Therefore, it should be used to describe network architecture rather than to prove molecular crosslinking mechanisms alone.

Scanning electron microscopy (SEM) is commonly used to observe dried gel surfaces and cross-sections, providing information on pore size, pore-wall thickness, wrinkles, aggregation, and network continuity [53,54]. Wang et al. showed that increasing Ca^2+^ concentration made sodium alginate/chitosan oligosaccharide beads more wrinkled and denser [37]. Chu et al. reported that SA/Tremella polysaccharide (TMP) hydrogels changed from loose lamellar structures to dense porous networks [11]. Zhu et al. found that starch derivatives produced more compact pectin/starch composite beads [50]. These studies show that SEM is useful for evaluating network densification.

SEM can also reveal defects caused by excessive crosslinking. Zhao et al. found that moderate Ca^2+^ produced smooth and dense cellulose nanocrystal-modified agar films, whereas excess Ca^2+^ caused roughness and aggregation [42]. Abbasi et al. showed that intermediate Ca^2+^ levels favored homogeneous κ-carrageenan/carboxymethylcellulose hydrogels, while higher levels disrupted uniformity [4]. Thus, SEM is valuable not only for showing reinforcement, but also for identifying optimal crosslinking windows.

The main limitation of SEM is that dehydration, drying, and coating may cause pore collapse, shrinkage, or rearrangement. Therefore, SEM images should not be directly regarded as the real hydrated structure of hydrogels. For high-moisture soft food systems, SEM should be interpreted together with cryo-scanning electron microscopy (Cryo-SEM), LF-NMR, rheology, and water-holding data.

Cryo-SEM is used to observe pore structure, wall thickness, and phase distribution under near-hydrated conditions, reducing artifacts from conventional drying [55]. El Hariri El Nokab et al. used Cryo-SEM to show that alginate network densification during aging occurred together with changes in the free-water fraction [25]. Liu et al. found that Ca^2+^-induced potato low-methoxyl pectin gels had lower porosity than acid-induced gels, consistent with higher storage modulus [18]. Cryo-SEM is therefore more suitable for linking hydrated microstructure with mechanical performance.

In encapsulation and composite gels, Cryo-SEM can show whether dispersed phases are embedded in the network. Wang et al. observed relatively uniform oil-droplet distribution in SA/COS gel beads [38]. Wang et al. visualized an interpenetrating structure between dense Alg/Ca^2+^ and porous casein networks [49]. However, freezing may still introduce ice-crystal damage, so Cryo-SEM should be cross-validated with confocal laser scanning microscopy (CLSM) or rheological data.

Atomic force microscopy (AFM) examines nanoscale aggregation, rearrangement, and surface structure, producing information on roughness, height profiles, chain conformation, and local microstructure [56]. He et al. showed that Ca^2+^ induced CMC self-assembly into a uniform network in CMC/gelatin films [43]. Jia et al. observed ring-like molecular-chain networks in Brasenia schreberi polysaccharide [33]. Yuan et al. found the densest alginate gel particles at 40 mmol/L Ca^2+^ [9]. AFM offers high resolution, but its small observation area cannot represent the whole three-dimensional gel network.

CLSM is suitable for analyzing component distribution, phase separation, and encapsulant location in composite gels, with outputs based on fluorescence-labeled spatial patterns [57]. Feng et al. observed the most uniform protein, polysaccharide, and lipid distribution in G4C4 whey protein–pectin gels [58]. Min et al. showed that different calcium salts altered phase separation in mung bean starch/flaxseed protein gels [6]. Rao et al. confirmed relatively uniform bacterial distribution in sodium alginate–pectin–whey protein system (SPW) probiotic capsules [52]. CLSM is therefore especially useful for evaluating spatial compatibility in composite polysaccharide-based systems.

The main limitation of CLSM is its dependence on dye selection, labeling efficiency, and image-threshold settings. Channel overlap or dye migration may also occur in multicomponent systems. Therefore, CLSM can support conclusions about phase distribution, but it cannot independently prove Ca^2+^ crosslinking mechanisms.

### 3.2. Spectroscopic Resolution of Intermolecular Interactions

Spectroscopic and scattering techniques mainly answer two questions: whether Ca^2+^ changes the chemical environment of functional groups on polysaccharide chains, and whether these changes lead to molecular ordering, crystallinity variation, or nanoscale network rearrangement. Compared with imaging methods, these techniques provide information closer to the molecular scale, but most signals remain indirect. Therefore, they should be combined with rheology, microscopy, and hydration analysis rather than used alone as final proof of Ca^2+^ coordination mechanisms.

FTIR is the most widely used method for identifying functional-group changes induced by Ca^2+^, with outputs including shifts or intensity changes in carboxyl, sulfate, hydroxyl, or amide bands. Zhao et al. used FTIR and ζ-potential analysis to show that Ca^2+^-mediated electrostatic interactions drove PPI/HMP complex formation [5]. Wang et al. confirmed Ca^2+^ crosslinking with SA carboxyl groups and electrostatic interactions between SA and COS in SA/COS gel beads [37]. Abbasi et al. observed carboxyl peak shifts in KC/CMC hydrogels, indicating ionic coordination [4]. Chu et al. reported weakened hydrophilic-group signals after Ca^2+^ crosslinking in SA/TMP hydrogels [11]. These studies show that FTIR is useful for identifying involved functional groups, but it cannot independently determine coordination geometry or binding stoichiometry.

FTIR can also distinguish the relative contribution of Ca^2+^ crosslinking and other non-covalent interactions. Zhao et al. found that, in apple HMP, increasing esterification reduced carboxyl availability and weakened Ca^2+^ crosslinking, making hydrogen bonding and hydrophobic interactions more important [20]. Zhang et al. showed that a higher RG-I side-chain proportion in citrus pectin emulsion gels enhanced hydrogen bonding [19]. Thus, FTIR is valuable not only for detecting Ca^2+^ participation, but also for showing when hydrogen bonding or hydrophobic interactions become dominant stabilizing forces.

Wide-angle X-ray scattering (XRD) mainly evaluates whether Ca^2+^ crosslinking changes crystalline or amorphous states, with outputs such as peak disappearance, intensity changes, or amorphization [59]. Zhao et al. found that characteristic curcumin peaks disappeared in Ca^2+^-crosslinked PPI-HMP systems, indicating amorphous dispersion [5]. Chen et al. reported an amorphous structure in SA/KGM composite microspheres after crosslinking [39]. Chu et al. observed increased crystallinity and more ordered molecular arrangement after TMP incorporation into SA/TMP hydrogels [11]. Zhu et al. found that starch derivatives reduced crystallinity but improved thermal stability in pectin/starch gel beads [50]. These results suggest that XRD reflects ordering changes, but higher crystallinity does not necessarily mean better gel performance.

The limitation of XRD is that it is more suitable for crystalline or semi-crystalline domains than for highly hydrated amorphous gel networks. Ca^2+^-induced ionic crosslinking may strongly affect gel strength and water retention without producing clear diffraction peaks. Therefore, XRD should be treated as supporting evidence of ordering or amorphization, rather than a core indicator of crosslinking strength.

Small-angle X-ray scattering (SAXS) mainly addresses nanoscale network rearrangement, providing parameters such as fractal dimension, correlation length, pore features, and ordered-domain evolution [60]. Liu et al. used SAXS to compare acid-induced and Ca^2+^-induced potato LMP gels and found higher fractal dimension and lower porosity in the Ca^2+^-induced gels [18]. El Hariri El Nokab et al. combined SAXS/WAXS to show that aging promoted egg-box ordering and network densification in calcium alginate hydrogels [25]. These results indicate that SAXS is more suitable than SEM for explaining nanoscale structural differences related to macroscopic mechanics.

The strength of SAXS is its ability to move gel analysis from qualitative observation to semi-quantitative or quantitative structure description. However, its interpretation is model-dependent, and different fitting models may produce different parameters. SAXS should therefore be cross-validated with Cryo-SEM, rheology, and hydration data. It provides strong evidence for structure–property correlation, but is not a stand-alone mechanistic proof.

Raman spectroscopy complements FTIR by probing molecular vibrations, conformational changes, and encapsulant states. Cao et al. used Raman spectroscopy to verify lactoferrin encapsulation in alginate/gellan gum composite microspheres, and peak shifts indicated hydrogen bonding and electrostatic interactions [61]. Raman can be more sensitive to certain functional groups and active compounds, but it may be affected by fluorescence background, water, and sample heterogeneity. Therefore, it is best used as a complementary tool rather than as a replacement for FTIR.

NMR mainly addresses how polysaccharide fine structure and chain conformation affect Ca^2+^ responsiveness, providing information on monosaccharide composition, domain ratio, substitution degree, chain mobility, and conformation. Wu et al. used NMR to resolve the fine structure of LMP prepared by different degreasing methods and showed that HG length and RG-I ratio strongly determined gelation capacity [12]. Compared with FTIR, NMR is better suited to explaining why certain polysaccharides gel more readily, but it is more expensive, sample-demanding, and difficult to interpret in complex food matrices.

### 3.3. Physical Analysis of Hydration State and Thermal Stability

Hydration-state and thermal-stability analyses mainly address two questions: whether Ca^2+^-induced networks change the state of water in gels, and whether the resulting network improves structural stability during heating or storage. Unlike microscopy and spectroscopy, these methods focus more on the physical consequences of network formation. They cannot directly prove Ca^2+^ coordination mechanisms, but they can show how crosslinking affects water retention, network compactness, and thermal tolerance.

LF-NMR is widely used to evaluate water distribution in gels, with transverse relaxation time T_2_ and peak-area proportions as the main outputs. In general, shorter T_2_ values indicate bound or immobilized water, whereas longer T_2_ values represent free water in larger pores [62]. Liu et al. found a left shift of T_2_ in Ca^2+^-induced LMP gels, indicating reduced water mobility and a denser network [18]. Jiang et al. reported that increasing the SA proportion in lotus rhizome starch/SA hydrogels caused more water to become immobilized [10]. Zhang et al. also used LF-NMR to correlate water distribution with network structure in citrus pectin emulsion gels [19]. These studies show that LF-NMR is suitable for evaluating water restriction and water-holding capacity.

The value of LF-NMR lies in converting structural densification into quantifiable water-mobility information. Compared with SEM or Cryo-SEM, it does not directly show pore morphology, but it reflects the movement of water near pores and polymer chains. Therefore, LF-NMR is particularly useful when combined with rheology, water-holding capacity, and texture analysis. Its limitation is that T_2_ peak assignment is empirical, and the boundaries among bound, immobilized, and free water may differ across gel systems.

DSC can be used to analyze the proportions of freezable and non-freezable water, thermal transitions, and hydration state. El Hariri El Nokab et al. used DSC to quantify the ratio of free to bound water in calcium alginate hydrogels, providing supporting evidence for the relationship between hydration state and network structure [25]. Compared with LF-NMR, DSC is more suitable for identifying freezing behavior and thermal transitions than for monitoring water migration. Its advantage is the quantification of thermal behavior, but peak overlap and component transitions may complicate interpretation in complex food gels.

Thermogravimetric analysis (TGA) mainly evaluates mass loss and thermal decomposition during heating, with outputs such as initial degradation temperature, maximum decomposition temperature, and residual mass. Li et al. found that 5% CaCl_2_ treatment increased the decomposition temperature of SA/Lycium ruthenicum anthocyanin films, indicating improved thermal stability [63]. Chu et al. reported higher thermal stability in SA/TMP hydrogels after TMP incorporation [11]. Zhu et al. observed that starch derivatives reduced crystallinity but enhanced thermal stability in pectin/starch gel beads [50]. These findings suggest that thermal stability reflects overall network integrity rather than crystallinity alone.

The limitation of TGA is that testing temperatures are usually much higher than normal food processing or gastrointestinal conditions. Therefore, TGA results should not be directly equated with real eating stability. For texture-modified foods for older adults, TGA can indicate potential material stability during heating or storage, but it cannot replace cooking, freeze–thaw, oral-processing, or digestion-stability tests. Thus, TGA is better used as supporting evidence for structural stability rather than as direct evidence of food application performance.

### 3.4. Rheological Characterization of Ca^2+^-Induced Polysaccharide Matrices

Microscopy and spectroscopy describe structural features and molecular interactions, whereas rheology quantifies the mechanical response of the hydrated network. For highly hydrated, soft, and time-dependent Ca^2+^-induced polysaccharide hydrogels, SEM, FTIR, or XRD alone cannot determine whether a continuous stress-bearing network has formed. Rheology evaluates gelation kinetics, network strength, viscoelastic balance, flow stability, printability, and swallowing-related texture. Liu et al. related the higher storage modulus of Ca^2+^-induced potato low-methoxyl pectin gels to their lower porosity [18], while Lin et al. used real-time rheology to compare rapid sodium alginate gelation induced by different calcium sources [26]. Thus, rheology provides a key link between hydrated network structure and food functionality.

Dynamic oscillatory rheology is useful for analyzing the formation and stability of Ca^2+^-induced networks. Time sweep tests monitor changes in storage modulus G′ and loss modulus G″ during gelation, reflecting the rate of network formation. Frequency sweep tests indicate whether the system is elasticity- or viscosity-dominated. Generally, G′ higher than G″ with weak frequency dependence suggests a relatively stable gel network, whereas a higher loss tangent, tan δ, indicates weak-gel behavior or stronger viscous dissipation. Strain sweep tests define the linear viscoelastic region (LVR) and evaluate resistance to small deformation; a decrease in G′ beyond the LVR usually indicates structural disruption or yielding. Temperature sweep tests assess thermal stability and processing tolerance. Rivera-Hernández et al. used linear viscoelastic analysis to identify the dominant network contribution in low-acyl gellan/citrus pectin co-gels [40], and Zhao et al. showed that the thickening response of apple high-methoxyl pectin to Ca^2+^ depended on Ca^2+^ concentration and degree of esterification [20]. Therefore, Ca^2+^-crosslinked networks should be evaluated not only by final strength, but also by formation pathway, stability range, and failure threshold.

Steady shear rheology and yield behavior describe hydrogel performance during processing, extrusion, oral shear, and swallowing. Apparent viscosity and shear-thinning behavior are especially important for thickened fluids, which must remain stable at rest but flow under oral and pharyngeal shear. Yield stress is related to post-printing shape retention, bolus stability, and prevention of rapid liquid flow, while thixotropy and structural recovery indicate whether the network can rebuild after shear disruption. Tian et al. reported that calcium citrate improved the viscosity and stability of a rice-starch-based International Dysphagia Diet Standardisation Initiative (IDDSI) level 3 liquid system [64], and Charoensri et al. linked the shear-thinning behavior of Riceberry porridge with in vitro swallowing simulation [65]. In 3D printing, Liu et al. developed corn starch–hydrocolloid–Ca^2+^ edible inks that balanced shape retention with soft swallowing-compatible structures [7], while Jeong et al. showed that CaCl_2_ improved gel strength and printability in jelly systems [66]. These parameters are closer to real processing and eating conditions than a single hardness value.

Rheology also helps connect Ca^2+^ concentration, calcium salt type, release rate, polysaccharide fine structure, and composite-network architecture with food performance. Moderate increases in Ca^2+^ concentration often enhance G′, yield stress, and structural recovery, but excessive Ca^2+^ may cause local aggregation, phase separation, or brittleness. Thus, stronger rheological response does not necessarily mean better food performance. Min et al. found that different calcium salts changed the pore structure and IDDSI texture level of mung bean starch–flaxseed protein composite gels [6]. Israkarn et al. showed that gellan gum and calcium fortification altered the rheology of mung bean protein mixtures, suggesting possible synergistic or competitive effects in protein–polysaccharide systems [46]. Yan et al. reported that Ca^2+^ above a threshold weakened the elasticity and chewability of sorghum bran arabinoxylan/soy protein isolate mixed gels [47]. Han et al. developed pea protein–calcium alginate emulsion gels that combined IDDSI level 4–5 texture with vitamin D_3_ retention [67]. Thus, Ca^2+^ crosslinking density should be treated as a multi-objective design variable rather than simply a way to increase gel strength.

Rheology alone cannot prove Ca^2+^ coordination mechanisms, binding sites, or stoichiometry. Increases in G′ or yield stress, or decreases in tan δ, only indicate changes in the mechanical response of the hydrated network. They cannot distinguish direct coordination, ionic bridging, electrostatic screening, hydrogen-bond enhancement, or phase separation. Therefore, rheological data should be interpreted together with FTIR, ζ-potential analysis, SAXS, LF-NMR, SEM, or Cryo-SEM. Donati et al. revealed the stepwise and ordered nature of Ca^2+^ binding during pectate gelation [2], whereas El Hariri El Nokab et al. combined SAXS/WAXS, water-state analysis, and network aging to interpret calcium alginate hydrogels [25]. For geriatric foods, rheology should also be combined with oral processing, tribology, IDDSI testing, and sensory evaluation, because small-amplitude oscillatory rheology within the LVR cannot fully represent large-deformation events such as mastication, tongue–palate compression, saliva dilution, and swallowing.

To integrate the methodological information discussed above, Table 1 summarizes the main characterization techniques used for Ca^2+^-induced polysaccharide hydrogels, including their structural outputs, interpretive value, and evidence boundaries. This comparison highlights that no single technique can fully define the crosslinking mechanism or functional performance of these hydrogels; instead, multiscale and complementary evidence is required.

Based on this multiscale characterization framework, the functional performance of Ca^2+^-induced polysaccharide hydrogels can be more critically interpreted. Network compactness, pore structure, hydration state, and intermolecular interactions are not endpoints themselves, but structural variables that determine encapsulation efficiency, release behavior, texture modulation, digestibility, and probiotic protection. Therefore, the following section discusses how these structural features are translated into food-relevant functions.

## 4. Functional Properties of Calcium-Induced Polysaccharide Gels

### 4.1. Encapsulation, Protection, and Controlled Release of Bioactive Compounds

For Ca^2+^-induced polysaccharide gels used as delivery carriers, the central issue is not simply maximizing encapsulation efficiency, but balancing encapsulation, protection, and release. A higher crosslinking density can restrict diffusion and improve stability, but it may also reduce release efficiency and bioaccessibility. Therefore, these systems should be evaluated by how much they load, how well they protect, when they release, and whether the released compounds remain functional.

Encapsulation efficiency is governed by both physical retention within the network and molecular compatibility between the polysaccharide matrix and the bioactive compound. In pea protein–alginate double-crosslinked microgels, increasing CaCl_2_ from 0 to 0.20 g/L raised encapsulation efficiency from about 70% to 92.62% [68]. However, Lee et al. showed that for small molecules such as catechins, excessive crosslinking may trap the compound too strongly and reduce release efficiency [69]. Thus, high encapsulation efficiency should not be equated with high delivery efficiency.

Polysaccharide fine structure also strongly affects encapsulation, especially in pectin systems. LMP prepared by high-hydrostatic-pressure-assisted enzymatic treatment showed a stronger affinity for hydrophobic curcumin because of its higher RG-I proportion and branching degree, resulting in higher encapsulation efficiency than acid- or alkali-prepared LMP [70]. This finding indicates that encapsulation is controlled not only by Ca^2+^ crosslinking density, but also by domain composition and molecular compatibility. Carrier design should therefore match the polarity, size, and interaction mode of the target compound.

Protection mainly arises from two mechanisms: physical shielding by a dense network and molecular immobilization through hydrogen bonding, hydrophobic interactions, or electrostatic forces. Song et al. used GDL-controlled Ca^2+^ release to prepare sodium alginate coatings and significantly reduced oxygen permeability at 50% relative humidity [24]. Li et al. reported that 5% CaCl_2_ treatment reduced water-vapor permeability of SA/Lycium ruthenicum anthocyanin films by 74% [63]. These results show that Ca^2+^ crosslinking can improve barrier properties, but this effect depends on network homogeneity and environmental humidity.

Protective effects observed in static storage or digestion models should be interpreted cautiously. Geng et al. reported high bioaccessibility of vitamins C/E, but the conclusion was mainly based on a static digestion model and did not fully account for gastric emptying, pH gradients, intestinal shear, or food-matrix effects on gel integrity [71]. Therefore, high protection in model systems cannot be directly extrapolated to real gastrointestinal conditions. Dynamic digestion and real-food validation are needed.

Molecular immobilization is useful for improving the stability of small bioactive molecules. Li et al. developed calcium/chitosan double-crosslinked apricot polysaccharide hydrogels for anthocyanins, increasing loading by 174.73% and enhancing post-digestion antioxidant capacity by about six-fold [72]. Adding sweet potato protein hydrolysate during Ca^2+^-induced sodium alginate gelation increased anthocyanin encapsulation to 87.3% [73]. Liu et al. used calcium pectin microcapsules to encapsulate streptomyces lactate and reduced mango decay by 28% after 8 days [74]. These studies support composite immobilization strategies, but loading improvement should be distinguished from physiological effectiveness.

Enzyme immobilization further illustrates the evidence boundary of protection. Pectinase immobilized in calcium alginate beads retained more than 80% relative activity after seven reuse cycles [75], and lysozyme immobilized in calcium alginate–gelatin films reduced pathogens by 2.66–3.73 log CFU/mL [76]. However, these studies rarely evaluated whether immobilization altered enzyme conformation, catalytic efficiency, or substrate accessibility. Retained activity alone does not fully represent preserved catalytic performance.

Controlled release is mainly governed by crosslinking density, pore structure, and environmental responsiveness. Yuan et al. prepared alginate microgels with gradient crosslinking densities and found that the most densely crosslinked 40-Alginate (Alg) degraded most slowly during in vitro fermentation [9]. Wang et al. showed that increasing crosslinking density in casein–sodium alginate double-network gels improved water-holding capacity and hardness, thereby enhancing encapsulant protection [49]. Paiboon et al. compared internal and external gelation and found that different crosslinking routes produced different surface morphologies and release behavior [23]. Thus, Ca^2+^ dosage and release mode are key variables for controlling release rate.

Pectin fine structure provides a higher-level route for release design. Zhang et al. found that low-ester pectin with high RG-I and low molecular weight favored colonic release, whereas low-RG-I and high-molecular-weight pectin released more rapidly in the small intestine; high-ester pectin showed more gradual release throughout digestion [19]. Wu et al. further showed that only LMP with sufficient HG length and appropriate RG-I branching could form stable networks for effective release control [12]. Jia et al. compared pectins from different sources and found that citrus peel pectin, rich in HG and uronic acid, responded most strongly to Ca^2+^, whereas RG-I-dominated okra pectin showed weak gelation [63]. These studies indicate that release can be tuned by selecting polysaccharide source and structure, not only by adjusting Ca^2+^ concentration.

Environment-responsive release expands the application potential of Ca^2+^-polysaccharide gels. Liu et al. prepared gelatin/calcium alginate beads for resveratrol delivery, achieving 96.6% encapsulation efficiency and Korsmeyer–Peppas-type release in simulated intestinal fluid [77]. Yuan et al. developed κ-carrageenan/metal-ion anthocyanin gastric floating tablets, where Ca^2+^ improved hydrogel properties and prolonged gastric release to 300 min [78]. Zhu et al. used Pueraria starch–calcium alginate beads for puerarin delivery, with modified starch increasing encapsulation efficiency to 71.12% and enabling sustained release [79]. Zhang et al. constructed dual-network emulsion gels using coffee cherry-derived polysaccharides and calcium alginate; at a coffee cherry-derived polysaccharide (CCP)/SA mass ratio of 3:2, the gel showed strong resistance to gastric shear and increased curcumin bioaccessibility from 29.62% to 63.94% [80]. These systems show environmental responsiveness, but most evidence still comes from simulated digestion.

Recent studies have also considered the balance between Ca^2+^ release and nutrient absorption. Tian et al. found that alginate gels containing ≥1.08 mg/mL Ca^2+^ shrank and partially released calcium in the gastric phase, then disintegrated and released calcium in the intestinal phase, reducing alginate-related inhibition of calcium absorption [81]. Wu et al. showed that low-M/G alginate with 10 mM Ca^2+^ formed rigid egg-box structures that resisted gastrointestinal swelling and supported distal release [82]. Jin et al. found that a soy protein isolate adsorption layer regulated calcium alginate porosity and shifted β-carotene release from burst release to swelling-controlled release [83]. These findings indicate that delivery design should consider carrier stability, nutrient release, and mineral availability together.

### 4.2. Texture Regulation Functions

The value of Ca^2+^-induced polysaccharide gels in texture regulation lies not in simply increasing hardness or elasticity, but in adjusting crosslinking density, network uniformity, and water retention to meet specific processing, chewing, swallowing, or printing requirements. For texture-modified foods for older adults, an ideal gel should balance low hardness, suitable cohesiveness, good water retention, and oral safety. Therefore, texture evaluation should shift from whether a gel forms to whether it fits the intended food application.

Calcium salt type is a direct variable because different anions affect Ca^2+^ release, hydrogen-bonding capacity, and local pH. Min et al. compared calcium salts in mung bean starch/flaxseed protein composite gels and found that calcium gluconate, calcium lactate, and monocalcium phosphate produced different pore structures and different hardness, adhesiveness, cohesiveness, and gumminess [6]. Zhao et al. showed that the rheological response of apple high-methoxyl pectin to Ca^2+^ depended on both esterification degree and Ca^2+^ concentration [20]. Thus, choosing a calcium salt means choosing a gelation rate, network morphology, and texture mode.

Ca^2+^ treatment conditions also determine gel strength and structural uniformity. For kelp edible gel particles, suitable treatment conditions were CaCl_2_ temperature below 35 °C, 4 min treatment, and pH 8; deviations reduced gel strength [84]. In double-network systems, rapid Ca^2+^ release by CCIM produced a denser and more homogeneous structure than slow release by GIM [85]. Different calcium sources, including CaCO_3_, CaHPO_4_, and CaSO_4_, also affected the homogeneity and water-holding capacity of SPI–alginate double-network gels by changing Ca^2+^ release rate [86]. Therefore, Ca^2+^ should be treated as a kinetic and processing variable rather than only a concentration variable.

Oil or other dispersed phases can further change textural outcomes. In corn starch–alginate emulsion gels, oil content significantly affected gel strength, with 30% oil showing a favorable structure [87]. This indicates that texture regulation cannot be explained only by hydrocolloids and Ca^2+^. Lipids, proteins, or particulate fillers may strongly influence network continuity, phase distribution, mouthfeel, lubrication, and stability.

Polysaccharide type and ratio determine how Ca^2+^-mediated networks are reinforced. Zhao et al. compared polysaccharide additives in calcium sulfate-induced soy protein gels and found that curdlan increased elastic modulus mainly through hydrophobic interactions, KGM improved network uniformity through hydrogen bonding, and gellan gum acted more as a physical filler [3]. Chen et al. reported that increasing KGM in SA/KGM microspheres increased viscosity, enhanced network compactness, and reduced swelling [39]. These findings show that polysaccharides are not simple strengthening agents, but regulate texture through different interaction pathways.

Starch–polysaccharide systems show that texture regulation is often coupled with digestibility. Jiang et al. found that increasing SA in lotus rhizome starch/SA hydrogels enhanced network compactness and water binding, while improving starch digestion behavior [10]. Zhu et al. showed that starch derivatives increased internal crosslinking in pectin gel beads and improved water-holding capacity, hardness, springiness, chewiness, and gumminess [50]. Thus, texture should not be judged only by hardness or G′, because changes in network structure may also alter digestion.

Small-molecule additives may interfere with Ca^2+^ crosslinking and modify gel texture. Wan et al. found that sorbitol, xylitol, and erythritol inhibited low-methoxyl pectin–Ca^2+^ gelation through competitive coordination, hydrogen bonding, and water competition, with sorbitol showing the strongest inhibition [16]. Abbasi et al. also showed that moderate Ca^2+^ promoted homogeneous KC/CMC double-crosslinked networks, whereas excessive Ca^2+^ disrupted uniformity [4]. Therefore, low-sugar, low-calorie, or composite formulations must consider the side effects of additives on Ca^2+^ binding and water distribution.

3D printing extends Ca^2+^ texture regulation from gel-strength optimization to shape retention and swallowing adaptation. For printable hydrogel formulations, the target is not simply high gel strength, but a balance between flow during extrusion and self-support after deposition. Ideal inks should show shear-thinning behavior, moderate yield stress, rapid structural recovery, and sufficient storage modulus (G′) to maintain layer integrity. Jeong et al. found that ultrasonic treatment and CaCl_2_ addition both improved jelly printability, while CaCl_2_ further increased gel strength and yield stress [66], while Liu et al. showed that Ca^2+^ bridging with anionic hydrocolloids enhanced shape retention in edible inks for dysphagia foods [7]. However, excessive Ca^2+^ crosslinking may increase extrusion resistance, nozzle clogging, poor interlayer fusion, difficult oral breakdown, and swallowing risk. Therefore, Ca^2+^ concentration, calcium-release rate, and polysaccharide ratio should be optimized to balance printing fidelity with swallowing suitability. Yang et al. used high-ester apple pectin and xanthan gum as composite thickeners to maintain weak-gel properties under Ca^2+^ and acidic conditions, meeting the textural needs of easy-swallowing foods [88]. For dysphagia-friendly foods, the goal is not the strongest gel, but a soft structure that is printable, stable, and easy to break down orally.

### 4.3. Intestinal Microecological Regulation Function

The intestinal microecological function of Ca^2+^-induced polysaccharide gels should not be described simply as a “prebiotic” or “gut-health” effect. The key question is whether these gels can regulate substrate delivery site, fermentation rate, microbial response, metabolite production, and intestinal barrier protection. Current evidence is mainly derived from in vitro fermentation, simulated digestion, and limited animal models. Therefore, this section should emphasize potential regulatory mechanisms rather than confirmed health benefits.

The first strategy is to regulate polysaccharide fermentation rate and microbial structure through Ca^2+^ crosslinking density. Yuan et al. showed that higher crosslinking density in alginate microgels slowed in vitro fermentation and selectively enriched Bacteroidetes-related communities associated with the crosslinked structure [9]. This suggests that Ca^2+^ crosslinking can affect not only degradation rate, but also how dietary fibers are utilized in the colon. However, in vitro fermentation data cannot directly prove long-term gut microbiota remodeling in vivo.

Probiotic delivery systems further use Ca^2+^ networks to improve gastrointestinal tolerance. Rao et al. found that SA–pectin–whey protein wall materials formed dense structures that improved probiotic tolerance and release in simulated gastrointestinal fluids, with protective effects in a mouse model of ulcerative colitis [52]. This provides stronger functional evidence than release testing alone, but animal-model results should still be distinguished from real-food intake and human validation.

The second strategy is to regulate starch digestion through Ca^2+^-crosslinked networks, thereby indirectly affecting intestinal microbiota. Jiang et al. found that interpenetrating lotus rhizome starch/SA hydrogels reduced rapidly digestible starch and increased slowly digestible and resistant starch fractions [10]. Yang et al. reported that Ca^2+^ chelation with hydroxyl groups in black soybean RS3 increased resistant starch content from 9.70% to 22.26% [31]. These findings indicate that Ca^2+^ can regulate starch digestion through both physical barriers and molecular rearrangement, but downstream microbiota effects still require fermentation and in vivo evidence.

The third strategy is probiotic protection and intestinal release. Zhu et al. showed that Ca^2+^-crosslinked pectin beads promoted Lactobacillus plantarum Lp3a growth, especially after adding a starch–curcumin complex [50]. Chu et al. developed Alg–TMP–Ca^2+^ bilayer hydrogels that protected probiotics in the gastric phase, promoted intestinal release, and improved tolerance to bile salts and antibiotics [11]. Chen et al. encapsulated Lactobacillus plantarum in SA/KGM microspheres, with an encapsulation efficiency of 81.5% at an ALG/KGM ratio of 1:3 [39]. These studies show that Ca^2+^ gel networks are useful probiotic-protection matrices, but encapsulation efficiency and in vitro survival should not be equated with colonization or clinical benefit.

Composite wall materials can further improve probiotic delivery. Bai et al. optimized sodium alginate–calcium lactate–skim milk gels for Pediococcus acidilactici CCFM18, achieving 72.14% encapsulation efficiency and much higher viable release in simulated intestinal fluid than free cells [89]. Mgomi et al. used in situ cultivation to form Pediococcus pentosaceus biofilms inside calcium alginate beads, improving survival in simulated gastrointestinal fluids [90]. Yi et al. added pectin oligosaccharides to pea-protein-microgel-reinforced low-ester pectin beads, improving Lactobacillus rhamnosus survival in the upper gastrointestinal tract [91]. These strategies suggest that Ca^2+^ networks can synergize with proteins, dairy components, or prebiotics, but formulation complexity may increase challenges in scale-up and stability control.

Some studies use lipid filling or dual crosslinking to further enhance protection. Xie et al. introduced cocoa butter replacer into gelatin-based capsules and combined it with Ca^2+^ crosslinking to fill pores and improve Lactobacillus plantarum bioactivity [92]. Byeon et al. used transglutaminase and Ca^2+^ dual crosslinking to prepare gelatin/LMP microgels co-encapsulating probiotics and pectin oligosaccharides, showing good protection under simulated digestion, heating, and storage [93]. Wang et al. used pea protein–sodium alginate complexes with CaCl_2_ to encapsulate Lactobacillus plantarum and achieve gastric protection and intestinal release [68]. These results show that multiple networks can strengthen protection, but may also affect release timing, mouthfeel, and cost.

## 5. Calcium Ion-Induced Polysaccharide Gels in Geriatric Foods

**Figure 3:**
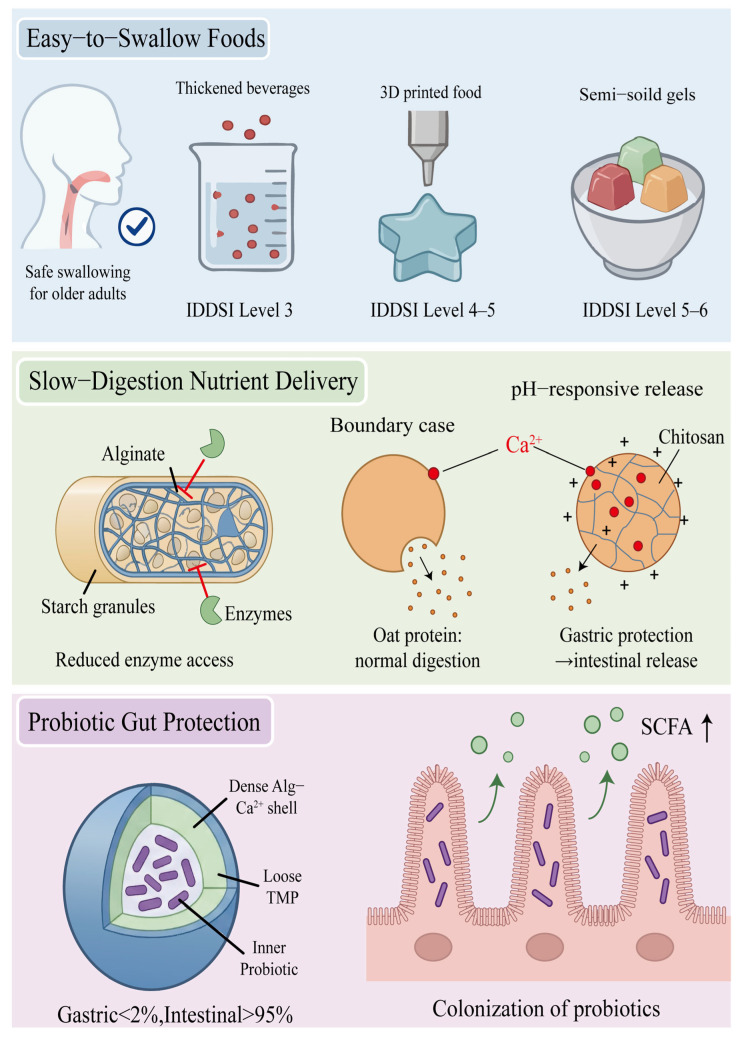
Application of Ca^2+^-induced polysaccharide hydrogels in geriatric food.

The application of Ca^2+^-induced polysaccharide hydrogels in geriatric foods is mainly based on three structure-derived functions: texture modulation, digestion-controlled nutrient release, and probiotic protection. As summarized in Figure 3, these applications are not independent endpoints, but are linked by the same structure–function logic. Ca^2+^-mediated crosslinking regulates network density, water distribution, pore structure, and mechanical behavior, which in turn determine swallowing suitability, nutrient-release profiles, and gastrointestinal protection.

Among these applications, texture modulation is the most direct route for developing easy-to-swallow foods. The following section discusses how Ca^2+^-induced polysaccharide gels can be used to regulate viscosity, softness, oral breakdown, and shape retention in liquid, semi-solid, and 3D-printed food systems.

### 5.1. Development of Easy-to-Swallow Foods Based on Texture Modulation

The purpose of easy-to-swallow food design is not simply to make foods softer, but to balance swallowing safety, oral processability, nutrient loading, and sensory acceptance. Older adults with dysphagia usually require foods with low firmness, low adhesiveness, suitable cohesiveness, and good water retention to reduce residue, aspiration risk, and insufficient intake [8]. The IDDSI framework provides a practical basis for texture classification, but IDDSI levels, instrumental texture, and real swallowing safety are not fully equivalent. Therefore, Ca^2+^-induced polysaccharide gels should be regarded as tunable texture-design tools rather than fully validated clinical solutions.

For liquid and thickened-fluid foods, the key challenge is viscosity stability and flow behavior during swallowing. Tian et al. showed that calcium citrate strengthened hydrogen-bonding networks among amylose, amylopectin, and water, reduced the radius of gyration of rice starch, and improved the viscosity and stability of rice-starch-based IDDSI level-3 fluids [64]. This indicates that calcium salts do not necessarily act through classical egg-box crosslinking in starch-based fluids, but may regulate molecular conformation and water organization through weak interactions. Such systems are useful for thickened beverages or liquid nutrition products, but direct swallowing-safety validation is still needed.

Compared with Ca^2+^ regulation, non-ionically crosslinked thickening provides another design route. Charoensri et al. reported that xanthan-gum-modified Riceberry porridge reached IDDSI level 4 at 0.5–2.0% addition, showed shear-thinning behavior, and correlated well with in vitro swallowing simulation [65]. The significance of this study is not to replace Ca^2+^ systems, but to show that easy-to-swallow foods can be designed through both hydrocolloid thickening and Ca^2+^-mediated structural regulation. In practice, the choice should depend on food matrix, target viscosity, thermal stability, and oral-residue risk.

For semi-solid foods, the design focus shifts from flow stability to the formation, breakdown, and water retention of soft networks. Min et al. compared calcium salts in mung bean starch/flaxseed protein composite gels and found that calcium gluconate and calcium lactate formed thin-walled porous and dense macroporous networks, respectively, both reaching IDDSI level 6, whereas monocalcium phosphate produced a lamellar brittle network and only reached IDDSI level 5 [6]. This shows that calcium salt anions can affect oral breakdown by modifying local pH, hydrogen-bonding capacity, and pore structure. For foods for older adults, calcium salt selection should serve the target texture level rather than simply maximize hardness.

Semi-solid gels can also function as nutrient-delivery matrices. Han et al. developed pea protein–calcium alginate emulsion gels that met IDDSI level 4–5 requirements and encapsulated fat-soluble vitamins [67]. Compared with simple starch–protein gels, emulsion gels provide both soft structure and lipid-phase nutrient loading. Thus, semi-solid foods for older adults should move from a single goal of safe swallowing toward the dual goal of safe swallowing and nutritional fortification.

3D printing further shifts easy-to-swallow food design from texture adjustment to personalized shape and precision nutrition. Liu et al. prepared 3D-printing inks close to IDDSI level 4 by combining Ca^2+^ ionic bridging with anionic hydrocolloids and starch gelatinization [7]. This system shows that Ca^2+^ crosslinking can improve post-printing shape retention while maintaining a soft structure suitable for swallowing. The main challenge is to coordinate printing accuracy, layer stability, and oral breakdown.

In 3D-printed systems, protein–polysaccharide emulsion gels offer stronger nutritional integration than simple starch-based inks. Han et al. used pea protein–calcium alginate emulsion gels for 3D printing, achieving 88.34% printing accuracy, IDDSI level 4–5 texture, and 86.35% vitamin D_3_ retention [67]. For geriatric nutrition, the value of 3D printing lies not only in shape customization, but also in texture grading, precise nutrient formulation, visual appeal, and the creation of small portions with high nutrient density. For older adults with dysphagia or reduced appetite, 3D printing can reconstruct pureed or homogenized foods into more recognizable and potentially more acceptable forms while maintaining IDDSI-compatible texture levels [7,67]. However, this technique also has limitations, including low production throughput, high equipment cost, narrow printable formulation windows, post-printing deformation, storage instability, and strict microbiological safety requirements. Current evidence remains mainly focused on printing accuracy, instrumental texture, and in vitro nutrient retention, whereas real mastication and swallowing performance, sensory acceptance, actual intake, and validation in older adults are still insufficiently studied.

### 5.2. Slow-Release Nutrient Delivery Based on Digestive Modulation

For older adults at risk of diabetes, metabolic syndrome, or impaired digestion, nutrient-delivery design should consider not only nutrient content, but also digestion rate, release site, and postprandial metabolic response. Ca^2+^-crosslinked polysaccharide gels can regulate the release of starch, minerals, and lipophilic nutrients through physical barriers, molecular chelation, interpenetrating networks, and pH-responsive behavior. Their main value is not simply to strengthen gels, but to control when and where nutrients are released and whether they remain bioaccessible.

Starch digestion is one of the most direct applications of Ca^2+^-induced polysaccharide gels. Jiang et al. showed that Ca^2+^-crosslinked lotus rhizome starch/sodium alginate hydrogels formed interpenetrating networks, reducing rapidly digestible starch by 26.9% and increasing resistant starch by 2.3-fold [10]. Yang et al. explained the molecular chelation route, showing that Ca^2+^ binding with hydroxyl groups in black soybean resistant starch increased RS3 content from 9.70% to 22.26% [31]. These two studies represent gel-barrier and molecular-rearrangement mechanisms, respectively, indicating that Ca^2+^ can regulate starch digestibility at different structural levels.

In alginate systems, the M/G ratio moves digestive modulation from simple crosslinking to structural-parameter design. Xie et al. found that in extruded buckwheat noodles, increasing the G-block proportion and crosslinking with 1% Ca^2+^ improved thermal stability, reduced cooking loss and elongation at break, and produced the lowest predicted glycemic index and highest resistant starch content when the SA M/G ratio was 1:2 with 1% Ca^2+^ [94]. Wang et al. introduced calcium alginate into pure buckwheat noodles and reduced glucose release by about 23.3 mg/g; XRD and FTIR showed that no new substances were formed, while the crystalline region of the noodles was altered [95]. Both studies support the role of calcium alginate in reducing starch accessibility, but extrusion and external addition may create different gel distributions and enzyme-access pathways.

The LMP-Ca^2+^ network provides another physical barrier based on pectin fine structure. Zhang et al. established a relationship between pectin structure and release rate by adjusting esterification degree and RG-I ratio [19]. Wu et al. further showed that stable LMP gelation requires a sufficiently long HG backbone and appropriate RG-I branching [12]. Jia et al. compared pectins from different sources and found that citrus peel pectin, with high HG proportion and uronic acid content, showed the strongest Ca^2+^ response [13]. These studies do not directly test starch digestion, but they provide the structural basis for understanding why LMP-Ca^2+^ barriers can be effective.

When LMP-Ca^2+^ barriers are applied to real foods, matrix type and processing route strongly affect digestive outcomes. Tan et al. added LMP to calcium-fortified wheat pasta and found that gel formation reduced water absorption, increased hardness, adhesiveness, and cohesiveness, and significantly lowered in vitro starch digestibility, with 2% LMP as the optimal level [96]. Kraithong et al. prepared LMP-Ca^2+^-embedded normal corn starch microspheres by electrospraying and found that 7% LMP produced the densest network, largest starch granule size, highest pasting temperature, and lowest starch hydrolysis rate [97]. Qin et al. encapsulated recrystallized starch in calcium alginate beads and evaluated its in vitro digestibility [98]. All studies support the barrier effect of LMP-Ca^2+^, but the different optimal LMP levels show that “best dosage” cannot be discussed without considering matrix and fabrication method.

Interpenetrating networks extend digestive barriers from surface coating to internal structural remodeling. The lotus rhizome starch/SA system showed that Ca^2+^-crosslinked SA and gelatinized or dextrinized starch entangled through hydrogen bonding, jointly restricting enzyme penetration and increasing resistant starch content [10]. Compared with simple coating, this strategy is more suitable for low-glycemic index (low-GI) staple foods because it forms a continuous internal barrier. Its limitation is that current evidence still relies mainly on in vitro starch hydrolysis and predicted GI rather than real postprandial glycemic response.

Slow-release delivery must also consider mineral release and absorption. Tian et al. found that alginate gels containing ≥1.08 mg/mL Ca^2+^ shrank and partially released calcium in the gastric phase, then disintegrated and released calcium in the intestinal phase, alleviating the inhibitory effect of alginate on calcium absorption [81]. Wu et al. showed that low-M/G alginate with 10 mM Ca^2+^ formed rigid egg-box structures that resisted gastrointestinal swelling and supported distal release [82]. These findings show that stronger networks do not necessarily ensure better nutritional effectiveness; release design must balance carrier stability with mineral bioaccessibility.

For lipophilic nutrients, Ca^2+^ gels often need to be combined with protein or lipid phases. Jin et al. introduced soy protein isolate adsorption layers into calcium alginate microgels, shifting β-carotene release from burst release to swelling-controlled release [83]. Han et al. developed pea protein–calcium alginate emulsion gels that met IDDSI level 4–5 texture requirements while encapsulating fat-soluble vitamins and improving vitamin D_3_ retention [67]. These systems integrate swallowing adaptation with nutrient delivery, but most evidence remains based on model foods and in vitro release.

### 5.3. Probiotic Delivery and Microecological Regulation Based on Gut Health

Older adults often experience reduced gut microbial diversity, increased harmful bacteria, weakened immunity, and a higher risk of low-grade inflammation. Therefore, intestinal-health-oriented foods for older adults should consider not only the number of probiotics added, but also their survival under gastric acid, bile, storage, and processing conditions, as well as their timely release in the intestine. Ca^2+^-induced polysaccharide gels are valuable because they can build protective networks, enable gastrointestinal-responsive release, and cooperate with prebiotics or proteins to improve cell stability. Their application should be positioned as a probiotic-delivery and gut-health-support platform rather than a fully validated clinical intervention.

Composite polysaccharide gels first address the limited protection offered by single-wall materials. Chen et al. compared sodium alginate alone with SA/KGM composite microspheres for Lactobacillus plantarum protection and found that the ALG/KGM ratio of 1:3 achieved 81.5% encapsulation efficiency while maintaining high viable counts after 40 min of gastric digestion [39]. In this system, Ca^2+^ mainly crosslinks alginate to form the skeleton, whereas KGM improves network compactness through viscosity enhancement, chain entanglement, and hydrogen bonding. This study shows that composite polysaccharides improve protection through cooperation between a primary ionic network and a secondary physical network.

Protein-filled composite wall materials can further enhance protection and provide preliminary evidence for intestinal barrier regulation. Rao et al. developed an SA–pectin–whey protein, SPW, system in which Ca^2+^ crosslinked both SA and pectin, while WPI filled network voids to form a denser wall; the system showed improved probiotic tolerance and release in simulated gastrointestinal fluids and protective effects in a mouse colitis model [52]. Compared with single calcium pectin beads, SPW benefits from both ionic crosslinking and protein filling. This provides a higher evidence level than in vitro release alone, although animal evidence cannot be directly translated into benefits for older adults.

The LMP/SA system reflects compatibility design between anionic polysaccharides. Zhang et al. optimized the LMP/SA ratio and CaCl_2_ concentration, achieving 87.69–99.98% encapsulation efficiency, viable counts above 7 log CFU/g after 4 h in simulated intestinal fluid, and similar levels after 30 d of storage at 25 °C [99]. Unlike SA/KGM, where a nonionic polysaccharide assists the ionic skeleton, LMP/SA involves two anionic polysaccharides that can both participate in Ca^2+^-mediated crosslinking. This system is suitable for emphasizing storage stability and intestinal release, but its long-term performance in real food matrices still needs validation.

For gastric protection and intestinal release, structural layering is more meaningful than simply increasing crosslinking density. Alg–TMP bilayer hydrogels use Ca^2+^-pre-crosslinked TMP as a loose inner layer for probiotic loading and Ca^2+^-crosslinked alginate as a dense outer shell, resulting in less than 1.99% Lactobacillus rhamnosus release in the gastric phase and more than 95% release in the intestinal phase [11]. The key innovation is the use of different inner and outer crosslinking densities to create pH-responsive release. Its limitation is that the integrity of the bilayer structure during heating, chewing, and real food storage remains unclear.

A simpler pH-sensitive strategy is provided by chitosan/sodium alginate/CaCl_2_ hydrogel beads. Liu et al. designed this monolayer system for rice bran bioactive peptide delivery; under gastric conditions, chitosan protonation induced network contraction, whereas intestinal pH promoted relaxation and release [100]. Although the cargo was not probiotic cells, this mechanism is relevant to probiotic protection because it separates gastric shielding from intestinal release. In parallel, Orellana-Palma et al. showed that calcium alginate and corn starch–calcium alginate beads stabilized freeze-concentrated sucrose/gallic acid solutions with Korsmeyer–Peppas-type release [101]. This study should be used as indirect evidence that starch–alginate networks may support water-soluble bioactive delivery and potentially provide fermentable matrix components, rather than as direct proof of probiotic efficacy.

Beyond conventional encapsulation, in situ biofilm formation provides another route to improve bacterial stress resistance. Mgomi et al. enabled Pediococcus pentosaceus to form biofilms inside calcium alginate beads, and the encapsulated cells showed significantly higher survival in simulated gastrointestinal fluids than conventionally encapsulated cells [90]. This strategy uses the bacteria’s own protective structure together with the gel network. However, the controllability, safety, and batch consistency of biofilm formation remain important challenges.

Prebiotic co-encapsulation links probiotic protection with substrate supply. Yi et al. added pectin oligosaccharides to pea-protein-microgel-reinforced low-ester pectin hydrogel beads, significantly improving the survival of Lactobacillus rhamnosus in the upper gastrointestinal tract [91]. This design is closer to a synbiotic strategy than a simple protective-wall approach, because pectin oligosaccharide (POS) may both regulate the matrix and provide fermentable substrates. Marinea et al. reported that oat protein digestion was not significantly influenced by pectin in either dispersion or gel systems [102]. However, the current evidence mainly focuses on survival, and further work should evaluate short-chain fatty acid production and microbial community changes.

Lipid filling can improve the barrier function of gel pores. Xie et al. introduced cocoa butter replacer into gelatin-based capsules and combined it with Ca^2+^ crosslinking to fill pores, significantly improving the bioactivity of Lactobacillus plantarum [92]. This strategy may strengthen hydrophobic barriers and gastric acid isolation, but it may also affect mouthfeel, fat content, and release rate. For foods for older adults, its influence on chewing, swallowing, and digestive burden should be considered.

Dual-crosslinked systems can improve stability during storage, heating, and digestion. Byeon et al. used transglutaminase and Ca^2+^ dual crosslinking to prepare gelatin/LMP microgels co-encapsulating lactic acid bacteria and pectin oligosaccharides, showing strong protection during simulated digestion, heating, and storage [93]. This system combines enzymatic protein crosslinking with ionic polysaccharide crosslinking, improving network strength and environmental tolerance. The trade-off is higher formulation and processing complexity, requiring a balance among cost, mouthfeel, and release controllability.

Protein–polysaccharide complexes can also support gastric protection and intestinal release. Wang et al. used pea protein–sodium alginate complexes with CaCl_2_ to encapsulate Lactobacillus plantarum, achieving gastric protection and intestinal release during digestion [68]. Protein components may improve interfacial stability and buffering capacity, but protein–polysaccharide ratio, addition sequence, and Ca^2+^ concentration strongly influence aggregation. This indicates that proteins are not passive fillers, but active regulators of interface structure and release behavior.

Dairy-component composite polysaccharide-based systems are closer to real food formulation. Bai et al. optimized sodium alginate–calcium lactate–skim milk gels for Pediococcus acidilactici CCFM18, achieving 72.14% encapsulation efficiency and much higher viable release in simulated intestinal fluid than free cells [89]. Skim milk may improve tolerance through protein buffering and network filling, while also improving food compatibility. Its limitations include possible allergenicity, flavor effects, and formulation-stability concerns.

Functional fillers can couple probiotic protection with bioactive-compound delivery. Zhu et al. found that Ca^2+^-crosslinked pectin beads promoted the growth of Lactobacillus plantarum Lp3a, and adding a starch–curcumin complex increased viable counts to 2.15 × 10^9^ CFU/mL, 2.92-fold higher than the blank group [50]. This study suggests that gel matrices can protect probiotics while improving the growth environment through bioactive fillers. However, increased viable counts in model systems should not be directly equated with improved in vivo colonization.

### 5.4. Translational Validation Framework for Geriatric Food Applications

Although Ca^2+^-induced polysaccharide hydrogels show potential for easy-to-swallow foods, controlled nutrient release, and probiotic protection, current evidence is still insufficient to demonstrate successful translation into geriatric foods. A more accurate position is that these systems have moved from model gels to real-food prototypes and food-like matrices, but most studies still provide evidence of structural feasibility, in vitro digestion behavior, or short-term functionality. Existing prototypes can be grouped into three categories: swallowing-oriented foods, including IDDSI-relevant thickened fluids, soft gels, and 3D-printed foods [6,7,64,67]; staple-food matrices, such as wheat noodles, buckwheat noodles, and starch microspheres, indicating that Ca^2+^-mediated networks can be incorporated into cereal-based real matrices [94,95,96,97,98]; and delivery-oriented food or food-like systems, such as protein–polysaccharide composite gels, dairy-component composite gels, and pH-responsive gel beads, which combine texture regulation with bioactive protection and intestinal release [89,99,100,101,102]. However, a real-food prototype is not equivalent to real eating effectiveness. IDDSI level, hardness, G′, or in vitro release profiles cannot replace swallowing imaging, sensory acceptance, meal intake, gastrointestinal tolerance, and long-term nutritional outcomes.

This evidence boundary is well recognized in dysphagia diet research. Thickened liquids may reduce aspiration under direct visualization, but whether this consistently improves clinical outcomes remains uncertain [103]. Modified-texture diets also involve informed consent, eating pleasure, quality of life, and long-term adherence [104]. Therefore, future studies on Ca^2+^-induced hydrogel foods should not only show that “a gel forms” or that “the matrix remains stable in the stomach”. They should answer three more relevant questions: whether older adults can swallow the food safely, whether they are willing to consume it repeatedly, and whether it improves nutrition- or health-related outcomes. Before such evidence is available, these hydrogels should be positioned as tunable platforms for geriatric food-structure design, rather than validated geriatric food solutions.

For digestion validation, the INFOGEST static model remains useful for standardized screening [105], but it cannot fully capture the dynamic behavior of Ca^2+^ hydrogels in the gastrointestinal tract, including gel fragmentation, Ca^2+^ dissociation, swelling or shrinkage, gastric emptying sequence, and nutrient-release kinetics. Dynamic gastrointestinal models should therefore become important tools in future validation. Dynamic stomach–small intestine systems such as DIDGI can simulate pH evolution, digestive secretion, gastrointestinal transit, and structural disintegration [106], whereas colon-fermentation models such as SHIME are suitable for evaluating fermentation rate, short-chain fatty acid production, and microbial changes after partially indigestible polysaccharides reach the colon [107]. For probiotic delivery or dietary-fiber systems, reporting survival or release in simulated gastrointestinal fluids is insufficient; colonic metabolism and microbial responses should also be assessed.

Organ-on-chip technologies can complement digestion models by addressing absorption, barrier function, and inflammation. Intestinal chips can incorporate microfluidic flow, peristalsis-like mechanical stimulation, epithelial barriers, mucus layers, immune components, and microbial co-culture [108]. For Ca^2+^ hydrogels, the more rational strategy is not to replace dynamic digestion models with chips, but to connect them sequentially. Dynamic gastric–small intestinal models can first generate relevant digesta fractions, which can then be introduced into intestinal chips to assess absorption, barrier integrity, inflammatory responses, and microbiota–host interactions. This approach provides more physiologically relevant evidence than a single in vitro release test, although chip systems still face limitations in standardization, throughput, and microbial stability. They should therefore be regarded as mechanistic platforms before human studies, not as substitutes for human evidence.

Computational modeling and machine learning may improve formulation design, but only when supported by high-quality data. Mechanistic models can simulate Ca^2+^ diffusion–crosslinking, nutrient release, intragastric mixing, particle breakdown, and gastric emptying. Existing computational studies of the stomach show that posture, gastric motility, and peristaltic patterns affect content distribution, dissolution, and emptying [109,110]. Thus, gastric stability of Ca^2+^ hydrogels should not be described only by residue at a single time point; it should be interpreted dynamically in relation to gel size, density, swelling behavior, and gastric motility. Machine learning may help predict relationships between formulation variables and IDDSI level, rheology, release rate, or sensory scores. However, if training data mainly come from small model-gel datasets, prediction may amplify existing bias. A more useful direction is to build standardized datasets integrating polysaccharide structure, calcium source, processing conditions, rheology, oral processing, dynamic digestion, and population acceptance.

Sensory and oral-processing evaluation remains a major weak point in geriatric food translation. Ca^2+^ crosslinking may improve shape retention, pregastric protection, and sustained release, but it may also increase hardness, adhesiveness, oral residue, dryness, and restricted flavor release. For older adults with impaired chewing or swallowing, a safe texture is not necessarily an acceptable texture. Future evaluation should combine IDDSI classification, shear rheology, extensional rheology, saliva dilution, oral breakdown, lubrication, flavor release, plate waste, and sensory scores from older adults. Extensional rheology is particularly relevant because boluses undergo not only shear but also stretching and breakup during oral-to-pharyngeal transport [111]. VFSS and deep-learning-based bolus segmentation can further quantify pharyngeal residue, aspiration risk, and bolus movement trajectories [112], and should be treated as key evidence for judging whether material-level texture modification translates into swallowing safety.

The trade-off between crosslinking density and functional performance should be central to Ca^2+^ hydrogel design for older adults. Lower crosslinking density generally favors softness, rapid oral breakdown, and immediate palatability, but weakens shape retention, thermal stability, and pregastric protection. Higher crosslinking density improves printability, gastric integrity, enzymatic resistance, and sustained release, but may increase hardness, adhesiveness, oropharyngeal residue, and eating fatigue. The goal should therefore not be maximum crosslinking, but an intermediate design window in which the food remains stable during processing and serving, breaks down safely in the oral phase, and releases nutrients or probiotics as intended during digestion.

Future studies should shift from material-performance indicators to care-relevant endpoints. Acute randomized crossover studies can compare Ca^2+^ hydrogel foods in terms of swallowing safety, pharyngeal residue, aspiration, meal intake, postprandial glucose or insulin response, and gastrointestinal tolerance. Short-term feasibility studies should assess actual intake, sensory acceptance, plate waste, constipation or bloating, and caregiver usability. Longer pragmatic studies should focus on body weight, nutritional status, muscle mass, quality of life, respiratory complications, hospitalization risk, and gut-microbiota changes. Only when material structure, real-food performance, dynamic digestion behavior, oral-processing safety, and population-level outcomes are connected can Ca^2+^-induced polysaccharide hydrogels move from promising food materials to evidence-supported design strategies for geriatric foods.

## 6. Conclusions and Perspectives

Ca^2+^-induced polysaccharide hydrogels provide a tunable platform for food structure design by linking molecular assembly, network architecture, and functional performance. In single-polysaccharide systems, Ca^2+^ induces gelation mainly through direct coordination, ionic bridging, charge screening, or helix stabilization. In composite systems, it further acts as a molecular bridge, competing ion, or network regulator in polysaccharide–polysaccharide, polysaccharide–protein, and multicomponent food matrices. Polysaccharide fine structure, calcium-release kinetics, pH, ionic strength, and processing conditions jointly determine crosslinking density, pore structure, hydration state, and mechanical behavior, thereby influencing controlled release, texture modulation, starch digestion, and probiotic protection.

Within this structure–function framework, rheology should be regarded as a central analytical tool rather than a supplementary characterization method. Imaging and spectroscopy can reveal network morphology and possible molecular interactions, but they cannot fully determine whether a hydrated gel forms a stable, deformable, and stress-bearing food matrix. Dynamic oscillatory measurements, steady shear tests, yield-stress analysis, thixotropic recovery, and thermal rheology are therefore needed to clarify how calcium source, calcium-release kinetics, polysaccharide fine structure, and composite formulation affect gelation behavior, network stability, oral breakdown, printability, and swallowing-related texture. Without this rheological evidence, conclusions based only on morphology or functional-group shifts remain insufficient for predicting real food performance.

For geriatric food, Ca^2+^-induced polysaccharide hydrogels show potential in easy-to-swallow foods, slow-release nutrient delivery, and intestinal health maintenance. By adjusting calcium salt type, polysaccharide composition, and processing route, soft foods with different IDDSI levels can be designed. By regulating M/G ratio, HG length, RG-I proportion, and interpenetrating network structure, starch digestion and nutrient release can be modulated. Through composite wall materials, layered structures, and pH-responsive networks, probiotic survival and intestinal release can be improved. However, these systems should be regarded as promising structure-design platforms rather than fully validated solutions for geriatric foods.

Important evidence gaps remain. Most studies are still based on model gels, static in vitro digestion, simulated gastrointestinal fluids, in vitro fermentation, or animal experiments, while validation in real food matrices, oral processing, sensory acceptance, long-term intake, and older-adult populations remains limited. Differences in polysaccharide source, molecular structure, calcium source, crosslinking condition, and evaluation method also hinder cross-study comparison. Future research should move from single-property evaluation toward a structure–function–population evidence chain. This evidence chain should include not only gel formation, instrumental texture, and in vitro release behavior, but also real-food matrix performance, oral-processing behavior, swallowing safety, sensory acceptance, actual intake, gastrointestinal tolerance, and population-level nutritional outcomes. Acute crossover studies, short-term feasibility studies, and longer pragmatic studies are needed to determine whether calcium-induced hydrogel foods can reduce residue or aspiration risk, maintain meal intake, improve nutrient bioaccessibility, support gastrointestinal comfort, and remain acceptable during repeated consumption. Dynamic gastrointestinal models, in situ multiscale characterization, real-food validation, and population-relevant endpoints should therefore be integrated to determine whether gel-structure changes truly improve swallowing safety, nutrient absorption, and intestinal health. Computational simulation, machine learning, and 3D printing may support structural prediction, personalized texture design, and industrial translation, but their use should be grounded in real-food performance and human-relevant evidence.

## Figures and Tables

**Table 1:** Structural characterization methods and evidence boundaries for Ca^2+^-induced polysaccharide hydrogels.

Method	Main Output	Supported Interpretation	Limitation	Refs.
SEM	Surface/cross-section morphology, pores, wrinkles, aggregation	Network densification, defects, or over-crosslinking	Drying may collapse pores; not the hydrated structure	[37,42,50,53,54]
Cryo-SEM	Pore structure, wall thickness, phase distribution	Links hydrated microstructure with mechanics, water retention, and release	Freezing may cause ice damage; needs validation	[18,25,38,55]
AFM	Roughness, height, chain conformation, nanostructure	Chain assembly and surface densification	Small field and surface bias limit bulk interpretation	[9,33,43,56]
CLSM	Fluorescent spatial distribution, phase continuity	Component compatibility and encapsulant distribution	Dye- and threshold-dependent; cannot prove crosslinking	[6,52,57,58]
FTIR/ζ-potential	Band shifts/intensity, ζ-potential	Functional-group involvement, electrostatics, H-bonding	Indirect; cannot define coordination geometry or stoichiometry	[4,19,20,37]
XRD	Diffraction peaks, crystallinity, amorphization	Structural ordering and active-compound dispersion	Limited for hydrated amorphous gels; not crosslinking strength	[5,11,39,50]
SAXS/WAXS	Fractal dimension, correlation length, nanopores, ordered domains	Links nanostructure with strength and porosity	Model-dependent; needs cross-validation	[18,25]
Raman	Vibrational bands, conformational shifts, peak shifts	H-bonding, electrostatics, and encapsulation state	Affected by fluorescence, water, and heterogeneity	[61]
NMR	Monosaccharides, HG/RG-I, substitution, chain mobility	Source- or modification-dependent gelation	Costly; difficult for complex food matrices	[12]
Rheology	G′, G″, tan δ, LVR, gel point, viscosity, yield stress, shear thinning, thixotropy, recovery	Gelation kinetics, viscoelastic strength, flow stability, and swallowing-related texture	Test-condition dependent; cannot prove Ca^2+^ coordination mechanism or sensory acceptance alone	[26,40]
LF-NMR	T_2_, peak areas, water distribution	Water retention, compactness, water mobility	Empirical peak assignment; limited cross-system comparison	[10,18,19]
DSC	Thermal transitions, freezing/melting, water fractions	Hydration state and water binding	Peak overlap complicates interpretation; not proof of crosslinking	[25]
TGA	Weight-loss temperatures, degradation peaks, residue	Heating or storage stability	High test temperatures; not eating stability	[11,50,63]

## Data Availability

All the data generated by this research have been included in the article. For any assistance, it is possible to contact the corresponding author.

## Abbreviations

The following abbreviations are used in this manuscript:

AFM	Atomic force microscopy
Alg	Alginate
Ca-EDTA	Calcium ethylenediaminetetraacetate
Ca^2+^	Calcium ion
CaCl_2_	Calcium chloride
CaCO_3_	Calcium carbonate
CaHPO_4_	Calcium hydrogen phosphate
CaSO_4_	Calcium sulfate
CCP	Coffee cherry-derived polysaccharide
CFU	Colony-forming unit
CLSM	Confocal laser scanning microscopy
CMC	Carboxymethylcellulose
COS	Chitosan oligosaccharide
Cryo-SEM	Cryo-scanning electron microscopy
DA	Degree of amidation
DE	Degree of esterification
DSC	Differential scanning calorimetry
FTIR	Fourier transform infrared spectroscopy
G	Guluronic acid block
GDL	Glucono-δ-lactone
GI	Glycemic index
GM	Alternating guluronic acid/mannuronic acid block
HG	Homogalacturonan
HMP	High-methoxyl pectin
IDDSI	International Dysphagia Diet Standardisation Initiative
IPN	Interpenetrating polymer network
ΙCar	Iota-carrageenan
KC	κ-Carrageenan
KGM	Konjac glucomannan
LF-NMR	Low-field nuclear magnetic resonance
LMP	Low-methoxyl pectin
M	Mannuronic acid block
M/G ratio	Mannuronic acid/guluronic acid ratio
NMR	Nuclear magnetic resonance
POS	Pectin oligosaccharide
PPI	Pea protein isolate
RG-I	Rhamnogalacturonan I
RG-II	Rhamnogalacturonan II
RS3	Retrograded resistant starch
SA	Sodium alginate
SAXS	Small-angle X-ray scattering
SEM	Scanning electron microscopy
SPI	Soy protein isolate
SPW	Sodium alginate–pectin–whey protein system
TEM	Transmission electron microscopy
TMP	Tremella polysaccharide
XRD	Wide-angle X-ray scattering
ζ-potential	Zeta potential

## References

[1] Hu C., Lu W., Mata A., Nishinari K., Fang Y. (2021). Ions-induced gelation of alginate: Mechanisms and applications. Int. J. Biol. Macromol..

[2] Donati I., Benegas J., Paoletti S. (2021). On the Molecular Mechanism of the Calcium-Induced Gelation of Pectate. Different Steps in the Binding of Calcium Ions by Pectate. Biomacromolecules.

[3] Zhao H., Chen J., Hemar Y., Cui B. (2020). Improvement of the rheological and textural properties of calcium sulfate-induced soy protein isolate gels by the incorporation of different polysaccharides. Food Chem..

[4] Abbasi Z., Esfandiari Z., Rostamabadi H. (2025). Postbiotic-loaded κ-carrageenan hydrogels double cross-linked with carboxymethyl cellulose and calcium ions. Food Hydrocoll..

[5] Guo Q., Su J., Xie W., Tu X., Yuan F., Mao L., Gao Y. (2020). Curcumin-loaded pea protein isolate-high methoxyl pectin complexes induced by calcium ions: Characterization, stability and in vitro digestibility. Food Hydrocoll..

[6] Min C., Yang Q., Pu H., Cao Y., Ma W., Kuang J., Huang J., Xiong Y. (2023). Textural characterization of calcium salts-induced mung bean starch-flaxseed protein composite gels as dysphagia food. Food Res. Int..

[7] Liu W., Hu H., Mcclements D., Jin Z., Chen L. (2025). Fabrication and characterization of edible inks for 3D printing of dysphagia foods based on corn starch stabilized by calcium ions and hydrocolloids. Food Hydrocoll..

[8] Li M., Ji Q., Cai Z., Zhang S., Shao J., Cai W., Liao H. (2025). Polysaccharide emulsion gels for dysphagia-friendly surimi-based diets: Development and application. Food Res. Int..

[9] Yuan D., Xiao W., Gao Z., Hu B., Jiang W., Li Y., Wu Y., Ni X. (2023). Modulating in vitro fecal fermentation behavior of sodium alginate by Ca^2+^ cross-linking. Food Res. Int..

[10] Jiang X., Li L., Yan J., Zhang L., Wang C., Lai B., Wu H. (2024). Characterization of hydrogel beads constructed from gelatinized lotus rhizome starch and sodium alginate by calcium cross-linking. Food Hydrocoll..

[11] Chu L., Deng Y., Zhang M., Chen J., Lian Y., Chen B., Xie L., Jiang Y. (2025). The characteristics of sodium alginate-tremella polysaccharide assembled hydrogel induced by calcium ion and its protective effect on *Lactobacillus rhamnosus*. Food Hydrocoll..

[12] Wu C., Qi J., Liao J., Liu Z., He C. (2024). Study on low methoxyl pectin (LMP) with varied molecular structures cross-linked with calcium inducing differences in the gel properties. Food Hydrocoll..

[13] Jia X., Li X., Shao Y., Xiao M., Li X., Sun Y., Liu Y., Guo Q., Goff H. (2026). Structural and rheological properties of four pectins and their application in yogurt. Food Hydrocoll..

[14] Pak U., Yu Y., Ning X., Ho C., Ji L., Mayo K.H., Zhou Y., Sun L. (2022). Comparative study of water-soluble polysaccharides isolated from leaves and roots of *Isatis indigotica* Fort. Int. J. Biol. Macromol..

[15] Hosseini S.S., Khodaiyan F., Kazemi M., Najari Z. (2019). Optimization and characterization of pectin extracted from sour orange peel by ultrasound assisted method. Int. J. Biol. Macromol..

[16] Wan L., Yang Z., Cai R., Pan S., Liu F., Pan S. (2021). Calcium-induced-gel properties for low methoxyl pectin in the presence of different sugar alcohols. Food Hydrocoll..

[17] Jiang G., Zhang Y., Luo D., Zhu S., Wang Y., Li W. (2025). Drying Model and Mechanism of Sugar Beet Pulp Based on Its Crosslinking with Ca^2+^ and Cu^2+^. Foods.

[18] Liu J., Lei D., Tang L., Zeng F., Guan Y., Wu Q., Li H. (2025). The influence of pH and calcium ions on the gelation of low methoxy pectin from potato. J. Food Sci..

[19] Zhang W., Liao J., Qi J. (2026). Modulating limonin release via pectin fine structure in Ca^2+^-citrus pectin emulsion gels. Food Res. Int..

[20] Zhao X., Zhou Y., Wu Z., Chen J., Zhou F., Zhao G. (2023). Thickening effects of Ca^2+^ on apple high-methoxyl pectin: Dependences on Ca^2+^ concentration and the degree of esterification. Food Hydrocoll..

[21] Zhang Y., Zhao T., Ma X., Liu Y., Liu X., Gong G., Huang L., Wang Z. (2025). Innovative modified pectins enriched with galactose: Preparation, characterization, and gel-based properties of curcumin-delivered hydrogel beads. Food Hydrocoll..

[22] Wei H., Qi J., Liao J., Zhuo T., Xiao R. (2025). Co-influence of the degree of esterification and degree of amidation on the properties of acid-sugar-calcium amidated pectin gels. Food Hydrocoll..

[23] Paiboon N., Surassmo S., Ruktanonchai U., Kappl M., Soottitantawat A. (2023). Internal gelation of alginate microparticle prepared by emulsification and microfluidic method: Effect of Ca-EDTA as a calcium source. Food Hydrocoll..

[24] Song H., Lee S., Han J. (2025). Enhanced oxygen barrier properties of sodium alginate coatings in humid environments: Ionic crosslinking of sodium alginate by calcium ions released from calcium hydrogen phosphate and calcium carbonate. Food Sci. Biotechnol..

[25] El Nokab M., Sayed J., De Witte F., Dewettinck K., Elshewy A., Zhang Z., Van Steenberge P., Wang T., Sebakhy K. (2024). A comparative analytical study for the different water pools present in alginate hydrogels: Qualitative vs. quantitative approaches. Food Hydrocoll..

[26] Lin Y., Xu J. (2026). Evaluation of rapid gelation of sodium alginate induced by different calcium sources through rheological and microstructural analysis. J. Food Meas. Charact..

[27] Wang Y., Li L., Liu J., Yan J., Wang C., Lai B., Dong Y., Wu H. (2025). Involvement of Anion-Specific Effects in Changes in the Gelation and Thermodynamic Properties of Calcium Alginate Hydrogel. Foods.

[28] Núñez-Santiago M., Pérez-López A., Espinosa-Solares T., Nicolás-Vázquez M., Laureano-López B. (2023). Sol-gel transition diagram and theoretical study of κ-carrageenan in the presence of calcium ions. LWT Food Sci. Technol..

[29] Ge Q., Rong S., Yin C., Mcclements D., Fu Q., Li Q., Han Y., Liu F., Wang S., Chen S. (2024). Calcium ions induced z-carrageenan-based gel-coating deposited on zein nanoparticles for encapsulating the curcumin. Food Chem..

[30] Cardenas-Alvarado A., Zubieta-Otero L., Rodríguez-García M., Morales-Sánchez E., Gaytán-Martínez M. (2025). Effect of calcium hydroxide nanoparticles on the physico-chemical properties of nixtamalized maize flours. Food Chem..

[31] Yang Q., Zhang X., Gu C., Li M., Hu X., Gao Y., Min Z., Zhang W., Wu W. (2024). The mediation mechanism of calcium ions on black bean type 3 resistant starch: Metabolomics, structure characteristics and digestibility. Food Chem..

[32] Zhao S., He X., Wang Y. (2025). Ultra-high pressure processing enhances structural, rheological and in vitro digestibility properties of rice starch-calcium gluconate complexes. Food Chem. X.

[33] Jia Z., Chen Y., Niu C., Xu Y., Chen Y. (2025). Structural Characterization, Rheology, Texture, and Potential Hypoglycemic Effect of Polysaccharides from *Brasenia schreberi*. Foods.

[34] Bao H., Zhou R., You S., Wu S., Wang Q., Cui S. (2018). Gelation mechanism of polysaccharides from *Auricularia auricula-judae*. Food Hydrocoll..

[35] Ge Y., Zhang Y., Peng T., Yang L., Li X., Wang C. (2025). Gelling properties and formation mechanism of blueberry pomace polysaccharide gels induced by calcium ions. Food Chem..

[36] Fei W., Rong L., Qi X., Lv X., Chen J., Wen H., Xie J. (2025). Chemical composition, rheological properties and calcium-induced gelation mechanism of *Premna microphylla* Turcz polysaccharide. Food Hydrocoll..

[37] Wang N., Tian J., Wang L., Song C., Wen C., Fu Y., Song S. (2024). Fabrication, characterization, and antibacterial properties of sodium alginate/chito-oligosaccharide gel beads. Food Hydrocoll..

[38] Wang N., Tian J., Wang X., Wen C., Ai C., Song S. (2025). Interactions between alginate in emulsion, chito-oligosaccharide, and calcium form gel beads to enhance the oxidative stability of krill oil. Eur. Food Res. Technol..

[39] Chen W., Zhao K., Zang J., Hao R., Liu B., Du H., Xu W. (2025). Survival Behavior of *Lactobacillus plantarum* Loaded in Alginate/Konjac Glucomannan Composite Beads Under Storage and Gastrointestinal Conditions. Food Sci. Nutr..

[40] Rivera-Hernández L., Chavarría-Hernández N., Tecante A., López-Ortega M., Cuellar M., Rodríguez-Hernández A. (2023). Mixed gels based on low acyl gellan and citrus pectin: A linear viscoelastic analysis. Food Hydrocoll..

[41] Zhang L., Shen B., Zheng C., Huang Y., Liang Y., Fei P., Chen J., Lai W. (2024). Chitosan/oxidized sodium alginate/Ca^2+^ hydrogels: Synthesis, characterization and adsorption properties. Food Hydrocoll..

[42] Zhao J., Liu T., Xia K., Liu X., Zhang X. (2022). Preparation and application of edible agar-based composite films modified by cellulose nanocrystals. Food Packag. Shelf Life.

[43] He B., Wang S., Lan P., Wang W., Zhu J. (2022). Topography and physical properties of carboxymethyl cellulose films assembled with calcium and gelatin at different temperature and humidity. Food Chem..

[44] Feng L., Wang Y., Liu T., Zhao C., Chen Y., Wang F., Bao Y., Zheng J. (2024). Pectin-based emulsion gels prepared by acidic and ionotropic methods for intestinal targeted delivery in vitro. Food Hydrocoll..

[45] Ren S., Du Y., Zhang S., Lou F., Wang Z., Yang H., Jiang L., Wang Z., Guo Z. (2025). Calcium salt-induced rice starch-soybean protein composite gels: Mechanism of influence of calcium salt type and concentration on gel properties. Food Chem..

[46] Israkarn K., Buathongjan C., Gamonpilas C., Methacanon P., Wisetsuwannaphum S. (2022). Effects of gellan gum and calcium fortification on the rheological properties of mung bean protein and gellan gum mixtures. J. Food Sci..

[47] Yan J., Yin L., Qu Y., Yan W., Zhang M., Su J., Jia X. (2022). Effect of calcium ions concentration on the properties and microstructures of doubly induced sorghum arabinoxylan/soy protein isolate mixed gels. Food Hydrocoll..

[48] Chen H., Zhou M., Xu Z., Dong X., Ding X., Zhou X., Cui P. (2025). Novel fava bean 11S nanofiber gels for sustained ergothioneine delivery: A calcium ion and κ-carrageenan approach. Food Hydrocoll..

[49] Wang J., Chen Z., Zhang W., Lei C., Li J., Hu X., Zhang F., Chen C. (2023). The physical and structural properties of acid–Ca^2+^ induced casein–alginate/Ca^2+^ double network gels. Int. J. Biol. Macromol..

[50] Zhu W., Bai R., Liang S., Yuan Y., Zhang B., Fauconnier M., Richel A., Zheng Z. (2026). Enhanced structural properties of calcium cross-linked pectin gel beads incorporated with starch derivatives: Preparation, characterization, and in vitro prebiotic activity. Food Hydrocoll..

[51] Du Y., Zhang S., Sheng L., Ma H., Xu F., Waterhouse G., Sun-Waterhouse D., Wu P. (2023). Food packaging films based on ionically crosslinked konjac glucomannan incorporating zein-pectin nanoparticle-stabilized corn germ oil-oregano oil Pickering emulsion. Food Chem..

[52] Rao Y., Deng J., Zhang C., Song Y., Liu L. (2025). Probiotics encapsulated by calcium pectin/chitosan-calcium pectin/sodium alginate-pectin-whey through biofilm-based microencapsulation strategy and their preventive effects on ulcerative colitis. Food Hydrocoll..

[53] Bhuiyan M., Yeasmen N., Orsat V. (2026). Food surface characterization by scanning electron microscopy and fractal analysis: A review. Food Chem..

[54] Miura M. (2013). Digital Image Analysis. J. Jpn. Soc. Food Sci. Technol. Nippon Shokuhin Kagaku Kogaku Kaishi.

[55] Kyomugasho C., Christiaens S., Van de Walle D., Van Loey A., Dewettinck K., Hendrickx M. (2016). Evaluation of cation-facilitated pectin-gel properties: Cryo-SEM visualisation and rheological properties. Food Hydrocoll..

[56] Funami T. (2010). Atomic Force Microscopy Imaging of Food Polysaccharides. Food Sci. Technol. Res..

[57] Bhuiyan H., Yeasmen N., Orsat V. (2025). Assessment of food component distribution and structure by confocal laser scanning microscopy: A review. J. Cereal Sci..

[58] Feng J., Liu D., Wang Z., Li C., Huang W., Liu S., Li Y. (2025). Interpenetrating network hydrogels loaded with nanostructured lipid carriers for curcumin delivery: Impact of dual crosslinking with genipin and calcium ions. Food Res. Int..

[59] Ukkunda N., Santhoshkumar P., Paranthaman R., Moses J. (2025). X-ray diffraction and its emerging applications in the food industry. Crit. Rev. Food Sci. Nutr..

[60] Olakanmi S., Karunakaran C., Jayas D. (2023). Applications of X-ray micro-computed tomography and small-angle X-ray scattering techniques in food systems: A concise review. J. Food Eng..

[61] Cao L., Li J., Parakhonskiy B., Skirtach A. (2024). Intestinal-specific oral delivery of lactoferrin with alginate-based composite and hybrid CaCO_3_-hydrogel beads. Food Chem..

[62] Wang C., Wang X., Liu C., Liu C. (2021). Application of LF-NMR to the characterization of camellia oil-loaded pickering emulsion fabricated by soy protein isolate. Food Hydrocoll..

[63] Li Y., Qiu C., Qi Y., Yan Y. (2025). Water Resistance Improvement of Sodium Alginate and Black Wolfberry Anthocyanins Based Films Treated by CaCl_2_ for Food Packaging. J. Oleo Sci..

[64] Tian X., Huang J., Li H., Zhang C., Li T., Pan Y. (2025). Effects of calcium citrate on the stability of rice starch in a Level 3 liquid system. Food Hydrocoll..

[65] Charoensri P., Aspinall S., Liu F., Kijroongrojana K. (2024). Rheological, textural, and swallowing characteristics of xanthan gum-modified Riceberry porridge for patients with dysphagia. J. Texture Stud..

[66] Jeong H., Lee C., Min S. (2024). Improvement of jelly 3D printing using ultrasound treatment and calcium chloride. Food Sci. Biotechnol..

[67] Han Q., Sheng G., Bai H., Xu X., Wei X., Zhou Q. (2026). Development of 3D-printed pea protein-based vitamin D3 carrier emulsion gel system with high stability and its optimization. Food Hydrocoll..

[68] Wang X., Chen S., Zhang X., Liang B., Li X., Li Y., Sun C. (2025). Double-crosslinked pea protein isolation-sodium alginate composite microgels embedding Lactobacillus plantarum: Effect of calcium chloride concentration. LWT Food Sci. Technol..

[69] Lee J., Lim D., Chung D., Lee H. (2022). Optimization and release characteristics of catechin-loaded calcium pectinate beads by internal gelation. Food Sci. Biotechnol..

[70] Cai R., Pan S., Li R., Xu X., Pan S., Liu F. (2022). Curcumin loading and colon release of pectin gel beads: Effect of different de-esterification method. Food Chem..

[71] Geng M., Li L., Tan X., Teng F., Li Y. (2024). W/O/W emulsion-filled sodium alginate hydrogel beads for co-encapsulation of vitamins C and E: Insights into the fabrication, lipolysis, and digestion behavior. Food Chem..

[72] Li X., Cheng X., Fan G., Wu C. (2025). Physicochemical and sustained-release properties of double-crosslinked apricot polysaccharides hydrogels encapsulating blueberry anthocyanins. Food Biosci..

[73] Zhang M., Feng Y., Xiao J., Sun C., Tu J., Niu L. (2024). Sweet potato protein hydrolysates solidified calcium-induced alginate gel for enhancing the encapsulation efficiency and long-term stability of purple sweet potato anthocyanins in beads. Food Chem. X.

[74] Liu W., Huang K., Tan Z., Wang C., Wen T., Huang L., Hang F., Xie C., Wang S., Li K. (2024). Application of nisin-embedded pectin microcapsules for ‘Guiqi’ mango fruit postharvest preservation. Food Packag. Shelf Life.

[75] Ishak N., Serri N., Samsudin H., Murad M. (2025). Impact of immobilized pectinase-alginate beads on physicochemical properties, antioxidant activity, and reusability in papaya juice processing. J. Food Sci..

[76] Zhang Q., Wu N., Yao Y., Chen S., Xu L., Tu Y., Zhao Y. (2025). Controlled fabrication, structure and property of lysozyme composite antibacterial film immobilized by calcium alginate-gelatin. Food Chem..

[77] Liu C., Zhang W., Chen J., Zheng X., Fu C., Wu X. (2025). Fabrication of pH-sensitive gelatin/sodium alginate composite microbeads for potential delivery of resveratrol. LWT Food Sci. Technol..

[78] Yuan K., Chiang Y., Li P., Chiang P. (2024). Physicochemical and release properties of anthocyanin gastric floating tablets colloidized with κ-carrageenan/metal ions. Food Hydrocoll..

[79] Zhu W., Liu M., Chi H., Li L., Zheng Z., Zhang B. (2025). Preparation, structural characterization, and controlled release of puerarin-loaded complex and gel beads based on native/modified kudzu starch. Food Chem..

[80] Zhang T., Zhang Y., Hao R., Huang D., Li H., Zhang B., Li G., Jiang Y., Li D. (2025). Dual network interpenetration emulsion gels based on alginate and coffee cherry-derived polysaccharides: Preparation, characterization, and curcumin delivery. Food Chem..

[81] Tian J., Wang N., Song C., Kong F., Wen C., Song S. (2025). Effects of calcium and alginate ratios on calcium alginate properties and calcium reberation during in vitro digestion. Food Res. Int..

[82] Wu L., Schroën K., Corstens M. (2024). Structural stability and release properties of emulsion-alginate beads under gastrointestinal conditions. Food Hydrocoll..

[83] Jin H., Wang L., Yang S., Wen J., Zhang Y., Jiang L., Sui X. (2023). Producing mixed-soy protein adsorption layers on alginate microgels to controlled-release 8-carotene. Food Res. Int..

[84] Wang Y., Li Y., Chen T., Fan L., Liu Z. (2025). Effect of Calcium Treatment Process on the Quality Characteristics of Kelp Edible Gel Particles. J. Food Process Preserv..

[85] Su C., Li D., Wang L. (2025). From micropores to mechanical strength: Fabrication and characterization of edible corn starch-sodium alginate double network hydrogels with Ca^2+^ cross-linking. Food Chem..

[86] Ye X., Zhao Y., Sun L., Xu Q., Pang Z., Li J., Li H., Liu X. (2025). Modulation of double network assembly via different calcium sources and GDL concentrations for simulating intramuscular fat. npj Sci. Food.

[87] Su C., Li D., Wang L., Wang Y. (2024). Development of corn starch-sodium alginate emulsion gels as animal fat substitute: Effect of oil concentration. Food Hydrocoll..

[88] Yang H., Chou L., Hua C. (2024). Effects of Calcium and pH on Rheological Thermal Resistance of Composite Xanthan Gum and High-Methoxyl Apple Pectin Matrices Featuring Dysphagia-Friendly Consistency. Foods.

[89] Bai R., Zheng Z., Qiu Z., Zhu W., Yuan Y., Liang S., Qiao Y. (2026). Encapsulation of *Pediococcus acidilactici* in Sodium Alginate-Calcium Lactate-Skim Milk Gel Beads: Characterization and Simulated Digestion Analysis. J. Food Sci..

[90] Mgomi F., Yuan L., Farooq R., Lu C., Yang Z. (2024). Survivability and characterization of the biofilm-like probiotic *Pediococcus pentosaceus* encapsulated in calcium alginate gel beads. Food Hydrocoll..

[91] Yi H., Kang Y., Chang Y. (2025). Structural, physicochemical, and in vitro digestion properties of microgel-reinforced synbiotic hydrogel beads filled with pectic oligosaccharides as a delivery system for *Limosilactobacillus reuteri*. Food Chem..

[92] Xie Y., Zhang K., Ma X., Zhang Y., Wu X., Ma L., Zou L., Liu W. (2025). *Lactobacillus plantarum* P9 encapsulated in gelatin/gellan gum microcapsules with lipid filling and Ca^2+^ crosslinking: Enhancing digestive vitality and storage stability. Food Biosci..

[93] Byeon J., Kang Y., Chang Y. (2023). Physicochemical and in vitro digestion properties of gelatin/low-methoxyl pectin synbiotic microgels co-encapsulating *Lacticaseibacillus casei* and pectic oligosaccharides via double-crosslinking with transglutaminase and calcium ions. Food Hydrocoll..

[94] Xie K., Xu X., Gao C., Wang Z., Meng L., Feng X., Tang X. (2025). A starch-sodium alginate interpenetrating network enhances the structure, texture, and starch digestibility of extruded buckwheat noodles: Regulatory effects of mannuronate/guluronate ratios and calcium ion concentrations. Food Chem..

[95] Wang H., Zhang J., Han L., Cao J., Yang J., Zhang Y., Hu B. (2023). Calcium ion regulation of sodium alginate in pure buckwheat noodles shown by in vitro simulated digestion. Front. Nutr..

[96] Tan M., Yuliarti O., Wong A., Wong J. (2024). Structural and in vitro starch digestion of wheat flour noodles by calcium mediated gelation of low methoxyl pectin. Food Struct. Neth..

[97] Kraithong S., Junejo S., Jiang Y., Zhang B., Huang Q. (2023). Effects of pectin-calcium matrices on controlling in vitro digestion of normal maize starch. Food Hydrocoll..

[98] Qin K., Zhang R., Qin W., Ji N., Qin Y., Dai L., Xiong L., Sun Q. (2023). Construction and In Vitro Digestibility of Recrystallized Starch Encapsulated in Calcium Alginate Beads. Foods.

[99] Zhang F., Wang R., Zhang L., Yan L., Jia Y., Yang J., Wang X., Lü X. (2023). Enhanced viability of probiotics in composite hydrogel beads. J. Food Eng..

[100] Liu X., Liu L., Huang F., Meng Y., Chen Y., Wang J., Wang S., Luo Y., Li J., Liang Y. (2025). pH-sensitive chitosan/sodium alginate/calcium chloride hydrogel beads for potential oral delivery of rice bran bioactive peptides. Food Chem..

[101] Orellana-Palma P., Macias-Bu L., Carvajal-Mena N., Petzold G., Guerra-Valle M. (2023). Encapsulation of Concentrated Solution Obtained by Block Freeze Concentration in Calcium Alginate and Corn Starch Calcium Alginate Hydrogel Beads. Gels.

[102] Marinea M., Lopez-Sanchez P., Ortiz D., Larsson K., Ström A. (2025). Oat protein in vitro digestion is not influenced by pectin in dispersion or gel systems. Food Hydrocoll..

[103] Makhnevich A., Perrin A., Talukder D., Liu Y., Izard S., Chiuzan C., D’Angelo S., Affoo R., Rogus-Pulia N., Sinvani L. (2024). Thick Liquids and Clinical Outcomes in Hospitalized Patients with Alzheimer Disease and Related Dementias and Dysphagia. JAMA Intern. Med..

[104] O’Keeffe S.T., Leslie P., Lazenby-Paterson T., McCurtin A., Collins L., Murray A., Smith A., Mulkerrin S., SPARC (2023). Informed or misinformed consent and use of modified texture diets in dysphagia. BMC Med. Ethics.

[105] Brodkorb A., Egger L., Alminger M., Alvito P., Assunção R., Ballance S., Bohn T., Bourlieu-Lacanal C., Boutrou R., Carrière F. (2019). INFOGEST static in vitro simulation of gastrointestinal food digestion. Nat. Protoc..

[106] Ménard O.D.P., Dupont D., Verhoeckx K., Cotter P., López-Expósito I., Kleiveland C., Lea T., Mackie A., Requena T., Swiatecka D., Wichers H. (2015). The DIDGI^®^ System. The Impact of Food Bioactives on Health: In Vitro and Ex Vivo Models.

[107] Van de Wiele T., Van den Abbeele P., Ossieur W., Possemiers S., Marzorati M., Verhoeckx K., Cotter P., López-Expósito I., Kleiveland C., Lea T., Mackie A., Requena T., Swiatecka D., Wichers H. (2015). The Simulator of the Human Intestinal Microbial Ecosystem (SHIME^®^). The Impact of Food Bioactives on Health: In Vitro and Ex Vivo Models.

[108] Pimenta J., Ribeiro R., Almeida R., Costa P.F., da Silva M.A., Pereira B. (2022). Organ-on-chip approaches for intestinal 3D in vitro modeling. Cell. Mol. Gastroenterol. Hepatol..

[109] Lee J.H., Kuhar S., Seo J.-H., Pasricha P.J., Mittal R. (2022). Computational modeling of drug dissolution in the human stomach: Effects of posture and gastroparesis on drug bioavailability. Phys. Fluids.

[110] Acharya S., Halder S., Kou W., Kahrilas P.J., Pandolfino J.E., Patankar N.A. (2022). A fully resolved multiphysics model of gastric peristalsis and bolus emptying in the upper gastrointestinal tract. Comput. Biol. Med..

[111] Marconati M., Ramaioli M. (2020). The role of extensional rheology in the oral phase of swallowing: An in vitro study. Food Funct..

[112] Park D., Kim Y., Kang H., Lee J., Choi J., Kim T., Lee S., Son S., Kim M., Kim I. (2024). PECI-Net: Bolus segmentation from video fluoroscopic swallowing study images using preprocessing ensemble and cascaded inference. Comput. Biol. Med..

